# Comparative analysis of mycotoxin, pesticide, and elemental content of Canarian craft and Spanish mainstream beers

**DOI:** 10.1016/j.toxrep.2023.03.003

**Published:** 2023-03-20

**Authors:** Pablo Alonso González, Eva Parga Dans, Iván de las Heras Tranche, Andrea Carolina Acosta-Dacal, Ángel Rodríguez Hernández, Ana Macías Montes, Manuel Zumbado Peña, Octavio Pérez Luzardo

**Affiliations:** a Institute of Natural Products and Agrobiology (IPNA-CSIC), Av. Astrofisico Francisco Sánchez, 3, 38206 San Cristóbal de La Laguna, Santa Cruz de Tenerife, Spain; b Independent researcher, Spain; c Toxicology Unit, Research Institute of Biomedical and Health Sciences (IUIBS), University of Las Palmas de Gran Canaria, Paseo Blas Cabrera s/n, Las Palmas de Gran Canaria 35016, Spain; d Toxicology Unit, Research Institute of Biomedical and Health Sciences (IUIBS), University of Las Palmas de Gran Canaria, Paseo Blas Cabrera s/n, Las Palmas de Gran Canaria 35016, Spain; & Spanish Biomedical Research Centre in Physiopathology of Obesity and Nutrition (CIBERObn), Madrid 28029, Spain

**Keywords:** Beer, Craft Beer, Spanish Beer, Mycotoxins, Pesticides, Elemental Composition

## Abstract

The number of craft breweries and the volume of craft beer produced globally is growing exponentially. However, little is known about their differences with mainstream beers regarding mycotoxin profile, pesticide and pollutant residues and elemental composition. Given that beer is one of the most consumed beverages worldwide, it is important to shed light on its toxicological profile. In this study, samples of 23 craft beers and 19 mainstream Spanish beers were collected to perform a comparative analysis including 8 mycotoxins, 225 pesticide residues and 50 POPs, and 50 elements. Mycotoxins were not detected in craft beers, while 100% of mainstream beers presented at least one mycotoxin. In contrast, craft beers contained higher average pesticide residues than their mainstream counterparts, although significant differences were only found in Mepiquat and Metrafenone content. No persistent organic pollutants were detected in any sample. The elemental composition presented differences between the two groups both in the concentration of elements and their hierarchy. In conclusion, the toxicological profile of all beers was safe and is unlikely to constitute a hazard to consumer health. Craft beers present significant differences from their mainstream counterparts in all the dimensions explored.

## Highlights

• 23 Canary craft beers and 19 mainstream Spanish beers in glass bottles were analyzed.
• Mycotoxins were not detected in craft beers, while 100% of mainstream beers presented one mycotoxin.
• Craft beers contained higher average pesticide residues than their mainstream counterparts.
• The elemental composition presented significant differences between the two groups.
• The toxicological profile of all beers was safe, but differences between craft and mainstream beers exist.

## Graphical Abstract

**Figure:**
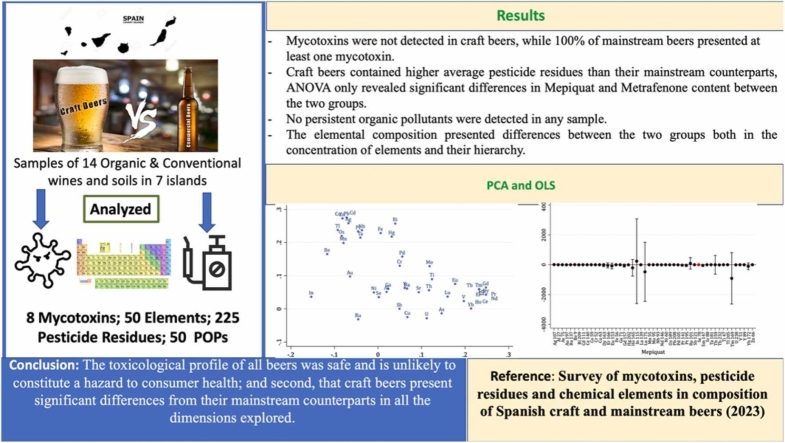

## 1 Introduction

Production of healthy and safe food is one of the main priorities in the European Union and around the world. Increasing consumer awareness about food pollutants and residues has led regulatory authorities to impose tighter monitoring on the quality of food products from production to consumption [5], [37]. This includes beer, one of the oldest fermented alcoholic beverages and the second most consumed in the world. Global beer consumption is led by China followed by the US and Brazil, although the highest per capita consumption occurs in European countries such as the Czech Republic, Austria or Germany, with more than 100 litres per year [10]. In 2020, Spain was the third overall beer producer in the EU after Germany and Poland, and the third in consumption after Germany and the UK, with a per capita consumption of 23 litres in 2020 [10], [38]. Like other EU countries, Spain has recently witnessed a surge in craft beers including ales that add to the former dominance of mainstream beers in the market. However, little is known about the toxicological profile of craft beers, especially when compared with their mainstream counterparts.

There is no uniform definition of craft beer globally, but their producers can generally be described as smaller, independent and traditional or innovative companies [39]. They can produce similar styles to mainstream companies (lager or pilsner), but most artisanal breweries produce different beer types such as stouts, ales, pale ales, porters, or wheat beers, with different alcohol contents. More than 10,000 craft breweries operate in the EU, with an exponential growth in recent decades thanks to their creative use of new ingredients, innovations in production methods and brewing steps, and in the creation of new flavours or the revival or traditional ones. These different procedures influence the toxicological profile of craft beers regarding pesticide residues, mycotoxins, and elemental composition. Craft producers do not generally microfiltrate, clarify or pasteurize, so they do not completely sterilize beers. Yeasts therefore remain in the bottle, making these beers prone to contamination [12]. They also use different raw materials, often of local origin to increase their links with the surrounding territory. Different malting techniques and unmalted adjunct cereals such as maize, rice, sorghum, and wheat, or even fruits, chocolate or coffee, also have an impact on both favour and composition in craft beers. Carbonation techniques often involve bottling the beer before fermentation ends, or adding sugar and yeast before bottling, instead of employing industrial carbonation methods.

The different characteristics of craft beers compared to mainstream beers require specific quality monitoring of various contaminants, including mycotoxins, pesticides and some chemical elements they contain [25]. Not only do these contaminants pose risks for human health, but they can also affect beer appearance, taste and the brewing process itself [63]. In particular, mycotoxin transfer from raw materials to craft beer has recently become a matter of concern in food safety, although no Maximum Residue Levels (MRLs) have been established for beer in the EU [48]. Mycotoxins are natural compounds produced as secondary metabolites of filamentous fungi, which appear given suitable environmental conditions and can cause disease [43]. Cereals used as raw material for beer production can be contaminated in various stages of production, mainly during storage. However, European legislation does not establish maximum mycotoxin levels in alcoholic beverages other than for OTA (2.0 µg/L) in wine [32]. For cereal based products such as beer, the regulation EC 1881/2006 [20] established maximum levels for 13 mycotoxins, including 2 µg/kg for aflatoxin B1 (AFB1) and 4 µg/kg for total aflatoxins (AFs), 750 µg/kg for Deoxynivalenol (DON), 75 µg/kg for Zearalenone (ZEN), 400 µg/kg for the sum of Fumonisin B_1_ (FB1) and Fumonisin B_2_ (FB2), and 5 µg/kg for Ochratoxin A (OTA) [46]. The presence of various types of mycotoxins in beers has been explored globally, in particular concerning OTA [54], [55]. Studies have recently focused on craft beers, given their rapid market growth, and show a higher percentage of such contaminants when compared with their mainstream counterparts [48]. In Spain, few studies have addressed mycotoxins in beers in general, and craft beers in particular [27], [36], [45], [46].

Pesticides and persistent organic pollutants (POPs) are some of the most toxic, mobile and environmentally stable elements that find various ways into the food chain. This due to their inherent chemistry, purpose or composition, (Rial-Berriel, Acosta-Dacal, Zumbado, Luzardo, 2020; [2]. Pesticide residues are a matter of concern in beer given their extensive use in cereal production and the growing consumer awareness about food contaminants (EFSA et al., 2021). Monitoring and reducing pesticide residues in beer is fundamental because pesticides can affect human health as well as beer brewing, impairing fermentation and microbial growth, modifying alcohol and polyphenol content, and other chemical parameters [31], [42], [41]. However, there are no specific MRLs set for pesticide residues in beer, therefore they derive from those stated for raw agricultural commodities such as barley or hops, taking into account changes in pesticide content due to processing techniques [22]. Much of the recent research has focused on experimental settings, rather than exploring commercially available beers by analysing the levels and fate of pesticides throughout the brewing process and in specific raw materials. Such studies show that the most pesticide concentrations are reduced during fermentation, pasteurization, clarification and filtration [18], [28], [41], [62]. These studies have shown that final pesticide levels are generally low [50]. Nonetheless, some studies have raised consumer concern by reporting pesticide levels at levels above the limit allowed in drinking water [16], [63], and 92% of beers on the Latvian market have been shown to contain glyphosate [30]. Most research has focused on mainstream beers. However, given that craft beers employ different brewing techniques, including avoidance of microfiltration and pasteurization, it can be expected that pesticide residues behave differently when compared with mainstream beers. To date, no studies address differences in levels of pesticide residues between commercially available craft and mainstream beers, and this remains a hitherto neglected topic in Spain, where the focus is on pesticide residues in wine [6].

The elemental composition of beer depends upon many factors. Endogenous chemical elements can come from the agricultural soil where raw materials are planted, the water employed, the cereal varieties, the environmental conditions, pesticides, and fertilizers applied to the soil [17]. Exogenous elements can pass on to the beer from the brewery equipment including pipes, tanks, filters or fermenters [47], [61], as well as from packaging such as kegs, casks or cans, which have shown to have a significant influence on beer composition, especially regarding aluminium content [9], [24], [29]. Various metals such as Hg, Pb, Cd, Al and As can pose health risks above certain concentrations, while others have an impact on beer quality and stability, including Cu, Fe and Mn. Another group can have positive or detrimental effects on human health depending on the dose, as is the case with Fe, Se or Zn [14], [51]. Elemental composition can also be used to discriminate the origin of beers [4]. However, few studies compare the elements contained in craft and mainstream beers, even less their correlation with pesticide and mycotoxin levels.

Given the scarce knowledge on craft beers in general, and their comparison with mainstream beers in particular, the aim of this paper is to explore the differences with a specific focus on Spain. It compares 23 craft beers with 19 mainstream Spanish beers, analysing 225 pesticide residues and 50 POPs, mycotoxins (AFB1, AFG1, DON, FB1, FB2, OTA, AFG_1_, AFG_2_ and toxins T-2 and HT-2), and their elemental composition including rare earths (50 elements in total). Owing to the huge number of craft beers in Spain, this paper focuses on craft beers exclusively from one region, the Canary Islands. They boast a lively craft beer market and a per capita consumption of 21.5 litres in 2020, being the ninth Spanish region in overall beer expenditure [38].

## 2 Material and methods

### 2.1 Beer samples

Forty-two beer samples were selected for analysis (see Table 1). Twenty-three craft beers produced and marketed in the Canary Islands were purchased directly from the breweries, comprising all the craft beers available in the Canary market when this study was carried out. The nineteen Spanish mainstream beers were acquired at local supermarkets, with the aim of obtaining a representative sample of the most widely available and consumed beers produced and marketed in Spain. The style of the mainstream beers was lager, except for one ale (S14). Substyles as reported in the label among mainstream beers included Dark (n = 1), Pale (n = 1), Red (n = 1), Marzen (n = 2), and Pilsner (n = 3). Craft beers were mostly ales, except for three lagers (S33, S34, S25). Substyles included Porter (n = 1), English Bitter (n = 1), Golden (n = 1), Sour (n = 1), Brown (n = 2), Pale (n = 4), Indian Pale (n = 1), Red (n = 1), Dry Stout (n = 1), Organic Blonde (n = 1), Witbier (n = 1), Berliner Weisse (n = 1), Black (n = 1) and Blonde (n = 2). Alcohol volume reported in the labels ranged from 4.5% to 8%. Sample containers were glass bottles of different capacities to avoid interference in the analyses. A code was assigned to each sample for analysis. Each bottle was opened to degas beer samples for at least 72 h. Then, 100 mL were collected in plastic containers and stored at − 20 C. Before each treatment, any residual gas was removed by subjecting the sample to ultrasonication for 30 min

**Table 1:** Sample list.

Number	Type	Beer style	Beer substyle
1	Mainstream	Lager	-
2	Mainstream	Lager	Marzen
3	Mainstream	Lager	Dark
4	Mainstream	lager	Pale
5	Mainstream	Lager	-
6	Mainstream	Lager	Pilsner
7	Mainstream	Lager	-
8	Mainstream	Lager	Amber
9	Mainstream	Lager	-
10	Mainstream	Lager	Pilsner
11	Mainstream	Lager	-
12	Mainstream	Lager	-
13	Mainstream	Lager	Marzen
14	Mainstream	Ale	Red
15	Mainstream	Lager	-
16	Mainstream	Lager	-
17	Mainstream	Lager	-
18	Mainstream	Lager	Pilsener
19	Mainstream	Lager	-
20	Craft	Ale	Porter
21	Craft	Ale	English Bitter
22	Craft	Ale	Golden
23	Craft	Ale	Sour
24	Craft	Ale	Brown
25	Craft	Lager	Pale
26	Craft	Ale	Indian Pale
27	Craft	Ale	Red
28	Craft	Ale	Dry Stout
29	Craft	Ale	Blonde (Eco)
30	Craft	Ale	Witbier
31	Craft	Ale	Berliner Weisse
32	Craft	Ale	Pale
33	Craft	Lager	Pale
34	Craft	Lager	Amber
35	Craft	Ale	-
36	Craft	Ale	Black
37	Craft	Ale	Blonde
38	Craft	Ale	-
39	Craft	Ale	Brown
40	Craft	Ale	Pale
41	Craft	Ale	-
42	Craft	Ale	-
43	Craft	Ale	Blonde

#### 2.1.1 Reagents, chemicals, and standards

Analytical-grade acetonitrile (ACN), methanol (MeOH), acetone (Ac), and formic acid (FA, HCOOH) were purchased from Honeywell (Morristown, NJ, USA). Nitric Acid (65% v/v) was acquired from Merck KGaA (Darmstadt, Germany). Ultrapure water was produced in the laboratory using a Milli-Q Gradient A10 apparatus (Millipore, Molsheim, France). Salts for extraction based on the AOAC QuEChERS method [33] were purchased from Agilent Technologies (Palo Alto, CA, USA).

All mycotoxin standards were supplied by Trilogy (Washington, USA), except zearalenone, employed as internal standard (IS), that was purchased from Sigma-Aldrich (Augsburg, Germany). Certified standard stock mix solutions of pesticides included in the multi-annual EU plan [15] were purchased from CPA Chem (Stara Zagora, Bulgaria) and individual certified standards of a selection of pesticides outside the programme (purity 95.19–99.9%) were acquired from Dr. Ehrenstorfer (Augsburg, Germany) and Sigma-Aldrich. Pure standards for all elements were purchased in acid solution (5% HNO_3_, 100 mg/L, CPA Chem, Stara Zagora, Bulgaria).

Working solutions were prepared for all the standards: a) mycotoxins at 1 µg/mL each in MeOH; b) pesticides, including POPs, at 0.833 µg/mL in ACN and; c) elements at 2 µg/mL each in 2% nitric acid.

#### 2.1.2 Sample preparation

##### 2.1.2.1 Mycotoxin analysis

Samples were prepared by direct dilution with ultrapure water in amber glass chromatographic vials (1:1, v/v). Prior to the analysis, 5 uL of the zearalenone IS working mix solution was added to each vial, and samples were mixed using a vortex. The calibration curve was prepared by adding the proper volume of mycotoxin working mixes to a mixture of beers that previously tested negative for any of the mycotoxins, diluted 1:1 (v/v) with water, as in the samples. The calibration curve covered the range 500–0.02 ng/mL and consisted of 12 levels. The same volume of IS mix was added to each point. This procedure was previously employed in our laboratory for different types of alcoholic beverages. When applied to beer samples, the entire procedure was validated for this matrix prior to use, using in-house fortified samples.

### 2.2 Pesticide and POP residues analyses

A method based on the QuEChERS technique [7] was used to extract the selected pesticides and POPs. The method was adapted from a previous development in our laboratory, after a full validation for beer matrix (Rial-Berriel et al., 2020, [1]. Quality Control samples (QCs), blanks and calibration curve were prepared in a beer that had been previously screened for the selected analytes, using the same methodology. The ten-point calibration curve covered the range 100–0.195 ng/mL and was prepared by adding the appropriate volume of working mix solution of pesticides and POPs to each tube. Similarly, QCs were prepared at a single concentration of 5 ng/mL. In the same step, 50 µl of P-IS mix solution was added to all samples, QCs, calibration points, and blanks and left to stand for 1 h in the dark, prior to extraction.

### 2.3 Elemental analysis

Beer samples were first subjected to vigorous agitation by Vortex. Afterwards, they were sonicated in ultrasonic equipment for 45 min to dissolve possible aggregates. Once sonicated, 1 mL of each was vigorously agitated again with Vortex to obtain a homogenized sample and placed in a digestion vessel with 8 mL of 65% concentrated ultrapure nitric acid. The samples were digested in a Milestone Ethos Up microwave oven (Ethos Up, Milestone SRL, Italy), as previously described [53]. To control recovery of the elements, 50 µl of the internal standard solution was added to each vessel. The digested sample was then diluted to a 15 mL with water and employed directly for subsequent determination of elements by ICP-MS. A reagent blank, prepared as for the samples, was included every 14 vials in the analytical batch.

The entire procedure was validated for beer prior to use, using in-house fortified samples. All determinations were performed in triplicate from each vessel, each vial being analysed three times in the ICP-MS. Therefore, for each beer sample, nine individual measurements were obtained. The recoveries ranged from 81% to 114% for toxic and essential elements. Linearity, Instrumental limits of detection (LOD) and quantification (LOQ), and Sample LOQ were calculated.

#### 2.3.1 Instrumental analyses

Analyses of pesticides, POPs, and mycotoxins were performed by gas and liquid chromatography coupled with triple quadrupole mass spectrometry (GC-MS/MS and LC-MS/MS) (Agilent Technologies, Palo Alto, USA). The retention times, precursor, fragment ions, and collision energies for each compound and equipment used for each compound are listed in the supplementary material in Tables S1 and S2. All the procedures have been previously described [35]; Rial-Berriel et al., 2020; [1].

The elemental analysis was performed using an Agilent 7900 ICP-MS (Agilent Technologies) equipped with standard nickel cones and a crossflow nebulizer with a make-up gas port (×400 nebulizer, Savillex Corporation, MN, USA) for all measurements. The entire procedure can also be found in previous publications [53].

### 2.4 Data analysis

Mean and standard deviations for each pesticide, mycotoxin, and element under analysis is presented. The software R (version 4.0.5) was employed to perform a first t-test analysis for each group to check if the difference between groups was statistically significant. Then, Stata (version SE 17) was employed to carry out analysis of variance (ANOVA) in all groups to explore differences in the concentrations between craft and mainstream beers. Finally, the relationship between pesticides with statistically significant differences and elemental composition was analysed through an OLS regression using robust standard errors that allows for correcting for heteroscedasticity. Principal Component Analysis (PCA) was employed to analyse the relationships between elemental components. This technique allows for reducing the dimensionality of the data by creating synthetic components that are linear functions of the original variables and explain most of the variability in our data.

## 3 Results and discussion

### 3.1 Mycotoxins

The most noteworthy finding was that craft beers showed no presence of the mycotoxins analysed (see Table 2). There are various potential explanations for this fact. First, none of the craft beers analysed employs adjunct cereals for brewing such as maize or rice, while their conventional counterparts in most cases do. Financial and logistic reasons explain the use of adjuncts by industrial breweries, such as better and more stable availability and the lower prices of these cereals [26]. However, adjunct cereals have the drawback of commonly presenting fungal contamination, leading to mycotoxin content. Second, craft beers make use of local cereals to a certain extent. Given the scarce cereal production in the islands and high costs, most craft beers include between 15% and 25% of local cereals, reducing or avoiding transport and storage periods that increase mycotoxin production [35]. Third, given the difficulties in importing large amounts of produce and regional customs system that hinders imports from mainland Spain and the European Union [5], craft beer producers in the Canary Islands do not generally keep large amounts of cereal in storage for long periods.

**Table 2:** Mycotoxin summary statistics.

	Category		
Component	Craft	Mainstream	Diff.
T2	0.00	0.01	-0.01
	(0.00)	(0.06)	[0.27]
Range	0.00–0.00	0.00–0.24	
FB2	0.00	10.05	-10.05 * **
	(0.00)	(7.15)	[0.00]
Range	0.00–0.00	0.00–29.55	
FB2	0.00	2.79	-2.79 * **
	(0.00)	(2.91)	[0.00]
Range	0.00–0.00	0.00–8.43	
DEOXY	0.00	0.18	-0.18 *
	(0.00)	(0.44)	[0.06]
Range	0.00–0.00	0.00–1.38	
AFG1	0.00	0.02	-0.02
	(0.00)	(0.08)	[0.27]
Range	0.00–0.00	0.00–0.33	
AFB1	0.00	0.03	-0.03
	(0.00)	(0.14)	[0.27]
Range	0.00–0.00	0.00–0.61	
N	24	19	
Occurrence = 0	24	0	
Occurrence = 1	0	9	
Occurrence > 2	0	4	

Column (1) shows the mean and standard deviation of the craft beers of the sample. Column (2) displays the same parameter with the beers categorized as mainstream. Column (3) illustrates the difference between craft and mainstream beers. Square brackets represent the p-value of the ANOVA specification which examines the existence of statistical differences between groups' means. N is the number of samples. Occurrence denotes the number of times a mycotoxin appears in a beer sample.

* ** p-value < 0.01, * * p-value < 0.05, * p-value < 0.1.

In contrast, 100% of the mainstream beers were contaminated with at least one mycotoxin and 47.36% of the analysed samples showed co-occurrence of mycotoxins (range 2–4). Thus, nine samples presented more than one mycotoxin, one sample had 4 mycotoxins, four samples 3, and four showed 2 mycotoxins. Nonetheless, all concentrations were well below the established limits. Among mainstream beers, OTA was not detected in any sample. Previous reports had found presence of OTA in Spanish beers although at very low levels [36], as in other European beers in general [55].

Mycotoxins T2, AFB1 and AFG1 were only found in one sample each. The T2 sample presented a low concentration level (0.24 μg/L). Low values and relatively low presence of T2 can be explained by the fact that only one wheat beer was included in our sample. Indeed, Rodríguez-Carrasco et al. [52] found higher levels between 24.2 and 38.2 μg/L in 14 of 154 beers analysed, all of wheat beer style. In turn, aflatoxin occurrence was low and at low levels in the same beer sample, with an AFB1concentration of 0.61 μg/L and 0.33 μg/L of AFG1. These results are in line with the low concentrations generally reported in the literature, between 0.1 and 3.7 μg/L [48], and with the rare aflatoxin contaminations reported in European beers [13]. Aflatoxins are considered as carcinogenic to humans (Group 1), and their consumption is of the highest toxicological concern.

Mycotoxin DON was detected in three different samples at low levels of 1.38, 1.30 and 0.65 μg/L. This goes counter to the literature, where craft beers often present higher concentration and occurrence of DON than their mainstream counterparts [48]. Concentrations found in our study are well below most reports in the literature, where contaminated samples present averages up to 63 μg/L [48], [60], ranging in Spain between 24.5 and 47.7 μg/L [52]. Our study further confirms the general agreement that DON is not a frequent contaminant of beer. For instance, both Bertuzzi et al. [8] and Varga et al. [60] did not find DON in the 106 and 374 beer samples they respectively surveyed.

Fumonisins were the most commonly found mycotoxins in our survey. All mainstream beers presented contamination with either FB1 or FB2. Specifically, FB1 was present in all samples but one, at concentrations ranging between 3.40 and 29.55 μg/L, while FB2 was present in 10 samples at between 4.02 and 8.43 μg/L. The occurrence of FB2 is significant, given that it was not detected by Peters (2017) in his survey of 1000 beers. There was co-occurrence of both fumonisins in 9 samples. Specific beer styles commonly reported as having higher fumonisin levels, such as dark or stout, do not present higher levels in our sample. The high prevalence of fumonisins in mainstream beers from Italy and Spain has been previously reported at levels of 30 and 85 μg/L respectively [8], [59], and generally explained by the use of rice and corn as adjunct cereals. Given that EFSA has set the toxicity levels of fumonisins by the sum of all their concentrations, the levels found in our study do not pose a risk. However, their high prevalence is a concern, since they are considered as possibly carcinogenic to humans and linked with oesophageal cancer [23].

#### 3.1.1 Pesticide residues

The analysis detected the presence of 15 pesticide residues out of the 225 tested, while no persistent organic pollutants were found among the 50 analysed. Only one sample was free from residues. This was a craft beer with organic certification, which was the only certified organic sample in the survey. The concentration of pesticide residues was overall low and well below the MRLs set by the European Union for barley and hops (See Table 3). Some pesticides such as Difeconazole, Triadimenon or Trifloxystrobin were only found in one or two samples at low levels. The pesticides with the highest occurrence were the systemic fungicide Boscalid (n = 40), the fungicide Mandipropamid (n = 32), the plant growth regulator Mepiquat (n = 32) and the systemic fungicide Dimethomorph (n = 25). Our results are partially aligned with previous research on the potentially riskiest category of pesticides. In this regard, Dušek et al. [18] analysed 58 pesticides in an experimental brewing setting, finding that thermostable pesticides were the most persistent and risky in beer production. Of those, our survey detected Azoxystrobin, Boscalid, Dimethomorph, Mandipropamid and Myclobutanil, but not Flonicamid, Imidacloprid or Thiamethoxam. Myclobutanil was also detected by [42], showing its potential influence on the fermentation rate and colour of young lager beers.

**Table 3:** Pesticide summary statistics.

	Category			
Component	Craft	Mainstream	Diff.	MRL
Azoxystrobin	0.71	0.29	0.42	1500 μg/L
	(1.59)	(0.24)	[0.27]	
*Range*	0.00–5.67	0.00–0.76		
Boscalid	19.06	18.80	0.26	4000 μg/kg
	(23.70)	(18.32)	[0.97]	
*Range*	0.00–86.06	0.00–52.76		
Chlorantraniprole	0.34	0.06	0.28	2000 μg/kg
	(1.37)	(0.25)	[0.39]	
*Range*	0.00–6.71	0.00–1.08		
Difenoconazole	0.02	0.00	0.02	3000 μg/kg
	(0.11)	(0.00)	[0.38]	
*Range*	0.00–0.53	0.00–0.00		
Dimethomorph	1.34	0.92	0.42	10 μg/kg
	(2.86)	(1.40)	[0.56]	
*Range*	0.00–13.03	0.00–6.02		
Dinocap	1.17	0.00	1.17	50 μg/kg
	(4.37)	(0.00)	[0.25]	
*Range*	0.00–20.28	0.00–0.00		
Flucythrinate	4.83	1.10	3.73	10 μg/kg
	(15.01)	(4.80)	[0.31]	
*Range*	0.00–59.32	0.00–20.93		
Fluopyram	0.14	0.09	0.05	200 μg/kg
	(0.24)	(0.22)	[0.43]	
*Range*	0.00–0.78	0.00–0.77		
Mandipropamid	1.17	1.68	-0.51	10 μg/kg
	(2.33)	(1.78)	[0.43]	
*Range*	0.00–8.61	0.00–6.21		
Mepiquat	3.02	1.32	1.70 *	40,000 μg/kg
	(3.48)	(2.39)	[0.08]	
*Range*	0.00–13.07	0.00–8.43		
Metrafenone	0.10	0.48	-0.38 * **	600 μg/kg
	(0.21)	(0.30)	[0.00]	
*Range*	0.00–0.82	0.00–0.95		
Myclobutanile	0.16	0.23	-0.07	10 μg/kg
	(0.34)	(0.21)	[0.46]	
*Range*	0.00–1.35	0.00–0.70		
Thiacloprid	0.03	0.00	0.03	900 μg/kg
	(0.14)	(0.00)	[0.38]	
*Range*	0.00–0.67	0.00–0.00		
Triadimenol	0.13	0.00	0.13	50 μg/kg
	(0.63)	(0.00)	[0.38]	
Range	0.00–3.11	0.00–0.00		
Trifloxystrobin	0.01	0.01	0.00	500 μg/kg
	(0.06)	(0.07)	[0.93]	
*Range*	0.00–0.31	0.00–0.28		
N	24	19		
Occurrence = 0	1	0		
Occurrence = 1	1	1		
Occurrence > 2	21	18		

Column (1) shows the mean and standard deviation of the craft beers. Column (2) displays the same parameter with the beers categorized as mainstream. Column (3) illustrates the difference between craft and mainstream beers. Square brackets represent the p-value of the ANOVA specification which examines the existence of statistical differences between groups' means. N is the number of samples. Occurrence denotes the number of times a pesticide appears in a beer sample. Given the lack of MRLs for beer, those have been retrieved from the EU database for barley. * ** p-value < 0.01, * * p-value < 0.05, * p-value < 0.1.

**Fig. 1:**
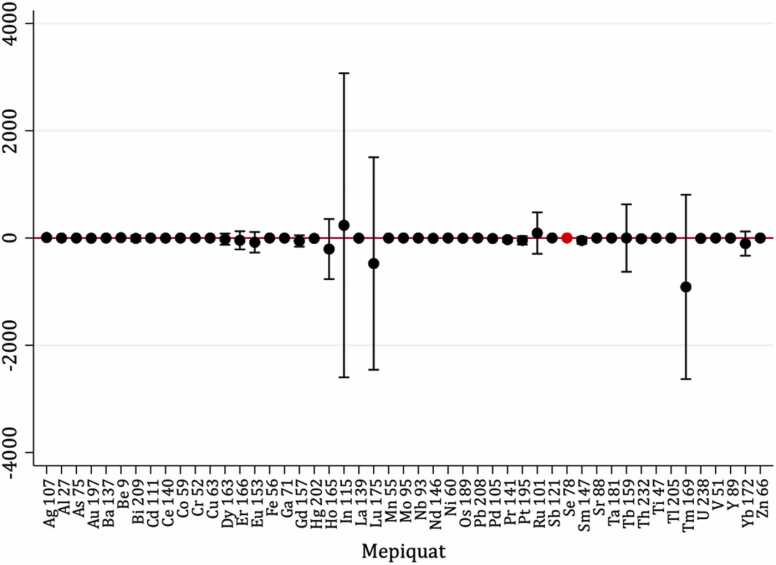
OLS regression to explore the relationship between a pesticide with statistically significant differences between beer types (Mepiquat) and elemental composition. This regression uses robust standard errors that allows for correcting for heteroscedasticity.

**Fig. 2:**
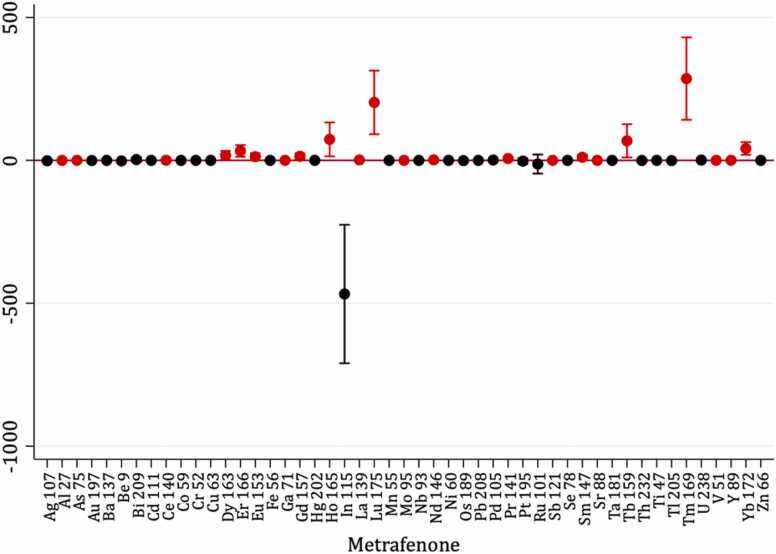
OLS regression to explore the relationship between a pesticide with statistically significant differences between beer types (Metrafenone) and elemental composition. This regression uses robust standard errors that allows for correcting for heteroscedasticity.

Craft beers presented higher average concentrations of all pesticides except Mandipropamid and Metrafenone. This can be explained by processing factors in mainstream brewing that can reduce and minimize the presence of pesticide residues, mostly brewing at higher temperatures, microfiltration and pasteurization [18], [28], [41]. However, the ANOVA only revealed significant differences in the higher levels of Mepiquat among craft beers, and, contrarily, in the higher levels of Metrafenone among mainstream beers (See Table 4). The origin of Mepiquat (1,1-dimethylpiperidinium) can be traced back as a residue from its application as a stem-stabilizing agrochemical in cereal crops, and also from its formation during the process of barley malting [64]. Mepiquat correlates with barley and beer colour, and has been recently found in 9 out of 10 commercial beers analysed in Canada [44]. Nonetheless, the levels found in our study are well below the European MRL and are not a regulatory concern in commercially available beers (See Fig. 1). In the case of Metrafenone (see Fig. 2), the average concentrations found were low (0.48 μg/L among the mainstream beers). This concentration is almost the same as the 0.5 μg/L in finished beers, reported as an average of the three processing factor studies presented in the EFSA evaluation for establishing new MRLs for Metrafenone [21].

**Table 4:** Elemental composition summary statistics.

	Category		
Component	Craft	Mainstream	Diff.
Zn 66	67.43	7.95	59.48 *
	(142.14)	(34.66)	[0.08]
*Range*	0.00–664.63	0.00–151.09	
Yb 172	0.00	0.01	-0.01 * **
	(0.00)	(0.00)	[0.01]
*Range*	0.00–0.01	0.00–0.01	
Y 89	0.03	0.14	-0.11 *
	(0.04)	(0.31)	[0.09]
*Range*	0.00–0.15	0.00–1.42	
V 51	1.40	41.69	-40.29 * **
	(3.35)	(36.67)	[0.00]
*Range*	0.00–16.65	0.01–137.05	
U 238	0.02	0.10	-0.08 * **
	(0.02)	(0.09)	[0.00]
*Range*	0.00–0.09	0.03–0.39	
Tm 169	0.00	0.00	0.00 * **
	(0.00)	(0.00)	[0.00]
*Range*	0.00–0.00	0.00–0.00	
Tl 205	0.05	0.00	0.05
	(0.12)	(0.00)	[0.10]
*Range*	0.00–0.37	0.00–0.00	
Ti 47	9.08	14.21	-5.13
	(8.98)	(19.37)	[0.26]
*Range*	0.00–28.07	3.48–76.90	
Th 232	0.01	0.01	0.00
	(0.02)	(0.01)	[0.92]
*Range*	0.00–0.08	0.00–0.04	
Tb 159	0.00	0.00	0.00 *
	(0.00)	(0.00)	[0.06]
*Range*	0.00–0.00	0.00–0.01	
Ta 181	2.23	1.66	0.57
	(2.65)	(1.37)	[0.40]
*Range*	0.00–11.02	0.37–4.50	
Sr 88	97.83	196.15	-98.32 * **
	(61.48)	(97.41)	[0.00]
*Range*	27.51–260.90	75.26–392.41	
Sn 118	0.00	0.00	0.00
	(0.00)	(0.00)	[.]
*Range*	0.00–0.00	0.00–0.00	
Sm 147	0.00	0.02	-0.02 * **
	(0.01)	(0.01)	[0.00]
*Range*	0.00–0.04	0.00–0.05	
Se 78	1.61	1.78	-0.17
	(1.27)	(1.34)	[0.68]
*Range*	0.00–4.98	0.00–4.74	
Sb 121	0.21	0.32	-0.11
	(0.37)	(0.52)	[0.43]
*Range*	0.00–1.90	0.00–1.73	
Ru 101	0.00	0.00	0.00
	(0.00)	(0.00)	[0.42]
*Range*	0.00–0.01	0.00–0.01	
Pt 195	0.00	0.00	0.00
	(0.01)	(0.00)	[0.89]
*Range*	0.00–0.04	0.00–0.02	
Pr 141	0.00	0.02	-0.02 * **
	(0.01)	(0.02)	[0.00]
*Range*	0.00–0.07	0.00–0.07	
Pd 105	0.04	0.07	-0.03 *
	(0.05)	(0.08)	[0.06]
*Range*	0.00–0.14	0.00–0.27	
Pb 208	2.12	0.00	2.12
	(6.79)	(0.00)	[0.18]
*Range*	0.00–32.73	0.00–0.00	
Os 189	0.04	0.00	0.04
	(0.10)	(0.00)	[0.15]
*Range*	0.00–0.42	0.00–0.00	
Ni 60	14.37	10.41	3.96
	(11.26)	(17.58)	[0.38]
*Range*	0.00–38.27	0.00–68.07	
Nd 146	0.02	0.08	-0.06 * **
	(0.05)	(0.07)	[0.00]
*Range*	0.00–0.23	0.00–0.27	
Nb 93	1.48	1.11	0.37
	(1.77)	(0.84)	[0.41]
*Range*	0.00–6.34	0.00–2.91	
Mo 95	3.28	6.55	-3.27 * **
	(3.81)	(4.11)	[0.01]
*Range*	0.33–14.56	1.02–16.42	
Mn 55	195.90	155.48	40.42
	(109.50)	(51.26)	[0.15]
*Range*	77.43–569.46	83.39–261.66	
Lu 175	0.00	0.00	0.00 * **
	(0.00)	(0.00)	[0.00]
*Range*	0.00–0.00	0.00–0.00	
La 139	0.02	0.07	-0.05 * **
	(0.05)	(0.08)	[0.01]
*Range*	0.00–0.22	0.00–0.26	
In 115	0.00	0.00	0.00 * **
	(0.00)	(0.00)	[0.00]
*Range*	0.00–0.00	0.00–0.00	
Ho 165	0.00	0.00	0.00 * **
	(0.00)	(0.00)	[0.00]
*Range*	0.00–0.00	0.00–0.01	
Hg 202	0.02	0.03	-0.01
	(0.07)	(0.10)	[0.62]
*Range*	0.00–0.26	0.00–0.32	
Gd 157	0.01	0.02	-0.01 * **
	(0.01)	(0.01)	[0.00]
*Range*	0.00–0.04	0.00–0.04	
Ga 71	0.05	0.13	-0.08
	(0.03)	(0.40)	[0.33]
*Range*	0.00–0.10	0.00–1.77	
Fe 56	60.86	49.58	11.28
	(142.60)	(121.44)	[0.78]
*Range*	0.00–603.49	0.00–385.32	
Eu 153	0.01	0.01	0.00 * *
	(0.01)	(0.01)	[0.02]
*Range*	0.00–0.02	0.01–0.04	
Er 166	0.00	0.01	-0.01 * **
	(0.00)	(0.01)	[0.00]
*Range*	0.00–0.02	0.00–0.02	
Dy 163	0.00	0.01	-0.01 * *
	(0.01)	(0.01)	[0.01]
*Range*	0.00–0.03	0.00–0.02	
Cu 63	41.67	56.36	-14.69 *
	(33.49)	(16.35)	[0.09]
*Range*	0.00–162.48	25.07–84.52	
Cr 52	2.28	3.50	-1.22
	(4.12)	(4.95)	[0.38]
*Range*	0.00–12.22	0.00–14.37	
Co 59	0.80	0.10	0.70 * *
	(1.43)	(0.12)	[0.04]
*Range*	0.09–7.12	0.00–0.33	
Ce 140	0.04	0.15	-0.11 * *
	(0.14)	(0.17)	[0.03]
*Range*	0.00–0.54	0.01–0.59	
Cd 111	0.26	0.03	0.23
	(1.06)	(0.08)	[0.34]
*Range*	0.00–5.23	0.00–0.37	
Bi 209	0.00	0.01	-0.01
	(0.01)	(0.01)	[0.21]
*Range*	0.00–0.07	0.00–0.04	
Be 9	0.07	0.00	0.07 * **
	(0.08)	(0.00)	[0.00]
*Range*	0.00–0.29	0.00–0.00	
Ba 137	27.74	33.20	-5.46
	(11.87)	(20.99)	[0.29]
*Range*	12.92–52.50	14.45–110.46	
Au 197	0.04	0.00	0.04
	(0.12)	(0.00)	[0.21]
*Range*	0.00–0.47	0.00–0.00	
As 75	1.01	3.20	-2.19 * **
	(0.71)	(1.60)	[0.00]
*Range*	0.27–3.56	0.49–6.50	
Al 27	19.76	628.98	-609.22
	(66.95)	(2347.12)	[0.21]
*Range*	0.00–239.28	5.74–10312.69	
Ag 107	0.02	0.00	0.02
	(0.05)	(0.00)	[0.23]
*Range*	0.00–0.23	0.00–0.01	

Column (1) shows the mean and standard deviation of the craft beers of the sample. Column (2) displays the same parameter with the beers categorized as mainstream. Column (3) illustrates the difference between craft and mainstream beers. Square brackets represent the p-value of the ANOVA specification which examines the existence of statistical differences between means. N is the number of samples. * ** p-value < 0.01, * * p-value < 0.05, * p-value < 0.1.

Although correlations between elemental composition and alcohol volume and other chemical parameters exist in the literature [17], no studies explore correlations between pesticide concentration and elemental composition. In this case, the analysis included Mepiquat and Metrafenone, to explore potential relations with elemental components (Table 5 and Fig. 1). The results reveal a positive and statistically significant association (at 95% confidence) between Mepiquat and Se. The negative relationship with other elements was discarded due to the impossibility of establishing a theoretical framework to explain how the presence of a pesticide can prevent the presence of some elemental components. In the case of Metrafenone (Table 5 and Fig. 2), more common in the mainstream group, the results show a positive relationship with more elemental components. Although explanation of these relationships goes beyond the scope of this paper, new research avenues could explore if other compounds in formulations including Mepiquat and Metrafenone contain the elements that are associated with them, or whether the presence of these pesticides affects the concentration of some elements during the brewing process.

**Table 5:** OLS results.

Component	Metrafenone	Mepiquat
Coefficient	(1)	(2)
Be 9	-1.77 * **	5.94
	(0.42)	(5.48)
Al 27	0.000042 * **	-0.00020 * **
	(0.0000053)	(0.000048)
Ti 47	0.0012	-0.040 * *
	(0.0020)	(0.017)
V 51	0.0048 * **	-0.0059
	(0.00099)	(0.022)
Cr 52	0.014	0.18
	(0.012)	(0.11)
Mn 55	-0.00017	0.0097
	(0.00045)	(0.0063)
Fe 56	-0.00031	-0.0024
	(0.00024)	(0.0026)
Co 59	-0.072 * *	-0.0078
	(0.031)	(0.14)
Ni 60	-0.0019	0.027
	(0.0034)	(0.037)
Cu 63	0.0029	-0.0024
	(0.0025)	(0.015)
Zn 66	-0.00056 * *	0.0022
	(0.00024)	(0.0025)
As 75	0.087 * **	-0.28
	(0.028)	(0.29)
Se 78	0.0021	0.97 * *
	(0.046)	(0.43)
Sr 88	0.0012 * **	-0.0082 * *
	(0.00043)	(0.0040)
Mo 95	0.027 * *	-0.065
	(0.013)	(0.13)
Ru 101	-12.9	91.6
	(16.6)	(191.6)
Pd 105	1.22 *	-8.96
	(0.63)	(5.76)
Ag 107	-1.43 * **	8.77
	(0.39)	(9.46)
Cd 111	-0.048 * **	0.0039
	(0.013)	(0.14)
Sb 121	0.24 * *	1.28 *
	(0.10)	(0.68)
Ba 137	0.0017	-0.0044
	(0.0019)	(0.026)
Os 189	-0.89 * **	-4.15 *
	(0.24)	(2.38)
Pt 195	-2.53	-44.0
	(5.07)	(38.4)
Au 197	-0.64 * **	-3.67 * *
	(0.12)	(1.56)
Hg 202	0.13	-6.94 * **
	(0.54)	(2.38)
Tl 205	-0.84 * **	0.55
	(0.16)	(3.67)
Pb 208	-0.010 * **	-0.00099
	(0.0029)	(0.026)
Bi 209	2.74	-6.96
	(4.08)	(28.1)
Th 232	-0.46	-13.6
	(2.13)	(20.4)
U 238	1.06	-7.71 *
	(0.69)	(4.19)
Ga 71	0.21 * **	-1.04 * **
	(0.049)	(0.38)
Y 89	0.43 * **	-1.98 * **
	(0.14)	(0.68)
Nb 93	-0.031	-0.28
	(0.025)	(0.26)
In 115	-467.5 * **	236.8
	(120.0)	(1403.5)
La 139	1.62 * *	-2.32
	(0.68)	(6.32)
Ce 140	0.69 * *	-1.50
	(0.32)	(2.61)
Pr 141	6.53 * *	-30.4
	(2.87)	(22.0)
Nd 146	1.84 * *	-6.42
	(0.80)	(6.20)
Sm 147	10.8 * *	-45.0
	(4.48)	(31.9)
Eu 153	12.7 * *	-80.1
	(6.20)	(94.7)
Gd 157	14.4 * *	-55.7
	(5.32)	(52.2)
Tb 159	68.3 * *	-0.64
	(28.8)	(311.2)
Dy 163	17.8 * *	-22.4
	(7.52)	(51.6)
Ho 165	73.5 * *	-206.2
	(29.4)	(277.1)
Er 166	33.2 * **	-43.4
	(10.2)	(83.8)
Tm 169	286.1 * **	-912.3
	(71.4)	(851.0)
Yb 172	41.5 * **	-103.9
	(11.0)	(111.7)
Lu 175	202.6 * **	-475.9
	(55.1)	(981.0)
Ta 181	-0.013	-0.18
	(0.017)	(0.17)
N	43	43

This table displays the relationship between the two pesticides with a statistical relationship and the presence of metals using an OLS estimation robust to heteroskedasticity. Column (1) uses Metrafenone as the dependent variable, while column (2) uses Mepiquat. * ** p-value < 0.01, * * p-value < 0.05, * p-value < 0.1 at 95% of confidence.

#### 3.1.2 Elemental composition

Differences in the presence of minor and trace elements (including metals and metalloids) were statistically significant between craft and mainstream beers. Also, there were different concentration orderings of each element according to method of production. Thus, the most abundant elements among craft beers were, in descending order: Mn > Sr > Zn > Fe > Cu > Ba > Al > Ni > Ti > Mo > Cr > Ta > Pb > Se > Nb > V > As > Co > Cd > Sb > Be > Ga > Tl > Ce > Os > Pd > Au > Y > Nd > Hg > La > U > Ag > Eu > Th > Gd. Undetected elements included: Yb, Tm, Tb, Sn, Sm, Ru, Pt, Pr, Lu, In, Ho, Er, Dy and Bi. Comparing mainstream beers, the ordering was as follows: Al > Sr > Mn > Cu > Fe > V > Ba > Ti > Ni > Zn > Mo > Cr > As > Se > Ta > Nb > Sb > Ce > Y > Ga > U > Co > Nd > Pd > La > Hg > Cd > Pr > Sm > Gd > Eu > Bi > Dy > Th > Er > Yb. Undetected elements included: Tm, Tl, Tb, Sn, Ru, Pt, Pb, Os, Lu, In, Ho, Be, Au and Ag. The average content of elements among the beers analysed was of different orders of magnitude. Among craft beers, only Mn, necessary for proper yeast growth, presented concentrations between 100 and 1000 μg/L. Zinc, Sr, Ni, Fe, Cu, Ba and Al ranged between 10 and 100 μg/L, V, Ti, Ta, Se, Pb, Nb, Mo, Cr and As between 1 and 10 μg/L, and Yb, Y, U, Tm, Tl, Th, Tb, Sn, Sm, Sb, Ru, Pt, Pr, Pd, Os, Nd, Lu, La, In, Ho, Hg, Gd, Ga, Eu, Er, Dy, Co, Ce, Cd, Bi, Be, Au and Ag between 0 and 1 μg/L.

In the case of mainstream beers, Sr, Mn and Al presented concentrations between 100 and 1000 μg/L, V, Ti, Ni, Fe, Cu and Ba between 10 and 100 μg/L, Zn, Ta, Se, Nb, Mo, Cr and As between 1 and 10 μg/L, and Yb, Y, U, Tm, Tl, Th, Tb, Sn, Sm, Sb, Ru, Pt, Pr, Pd, Pb, Os, Nd, Lu, La, In, Ho, Hg, Gd, Ga, Eu, Er, Dy, Co, Ce, Cd, Bi, Be, Au and Ag between 0 and 1 μg/L. Beyond differences in the concentrations and hierarchy of elements, ANOVA revealed significant differences between craft and mainstream beers in the case of Yb, V, U, Tm, Sr, Sm, Pr, Nd, Mo, La, Gd, Er, Be, and As (p-value < 0.01) and Eu, Dy, Co and Ce (p-value < 0.05), with Zn, Y, Tb, Pd and Cu, with a p-value < 0.1. These values are generally higher than those detected by Wyrzykowska et al. [66], who reported up to 200 μg/L of Rb, Mn and Fe, while our results only showed such high levels in the case of Mn. In their study, second-order metals (1–5 μg/L) included Cu, Zn, V, Cr, Sn, As, Pb, and Ni, which we generally found at different levels, both higher and lower. For instance, Cu, Ni and V were found in higher concentrations among both craft and mainstream beers, while others such as Sn were below 1 μg/L. However, the concentrations found align with the review of metals in beer by Pohl [51] and more recent reports on beer’s elemental composition [14], [24], [49].

In the case of Al, concentrations are in the ranges generally reported between 5 and 2200 μg/L, although one sample exceeds 10.000 μg/L despite being a glass bottled beer and not a can container. Ba levels lie in the range between 10 and 70 μg/L (craft =27.74 μg/L, mainstream=33.20 μg/L). Lower ranges were found in the case of Co, generally reported as high as 9.8 μg/L (craft=0.80, mainstream=0.10). Although it is difficult to discern between exogenous elements deriving from contamination and endogenous ones coming from raw materials, and from processing and storage, the origin of Co in beverages generally derives from cobalt powder employed in the processing of hard metal containers, enamels and pigments [66]. Low levels suggest good quality machinery and processing techniques being employed. Similarly, Cr, Cu, Fe, Mn, Ni, Pb, St and Zn are within the ranges reported in the literature (Bellido-Milla et al., 2000). As in the case of Co, the metals Fe and Zn are widely employed in the fabrication of containers for preventing corrosion, and their low levels indicate good quality machinery. Copper is employed in various plant protection formulations and can therefore be related with anthropogenic sources [34]. In the case of V, craft beers were in the range of 1–5 μg/L, but mainstream beers present much higher concentrations reaching 41.69 μg/L. Although the mechanism is not clear, accumulation of V can be inhibited by Mn, Ni and Cu in plants, which can explain different levels of V in mainstream beers [3]. As reported in the literature [11], [40], trace metals and rare earths such as Ag, Ga, Hg, U, and Sb were found at < 1 μg/L while others like In, Tl, Bi, and Th were at < 0.1 μg/L.

The toxicological profile of all samples was safe. Given that the EU has not set toxicological limits for metals in beer, the elemental composition of the samples was compared to the limits for drinking water established by the World Health Organization [65]. All elements of potential toxicological concern presented concentrations that were several orders of magnitude below the limits for drinking water. Thus, for Cd, the average concentration was 2.28 μg/L and 0.03 μg/L for craft and mainstream beers respectively (WHO limit of 0.003 mg/L). This is in line with reports ranging from not detected to 14.3 µg/L in Brazil [57] to 1 µg/L in Finnish beers [58] and 0.02–0.15 µg/L in the Czech Republic, and 0.16 µg/L in Italy [17]. The Scientific Cooperation (SCOOP) task 3.2.1113 also confirmed low Cd contents in 126 beer samples [19]. The average Pb concentration was 2.12 µg/L among craft beers, but this heavy metal was not detected among mainstream beers (WHO limit, 0.01 mg/L). Concentrations of Pb are low compared to those reported in Brazil, between 0 and 290 µg/L [57], 9 µg/L in Finland [58] and between 10 and 200 µg/L in the UK [56]. Other elements of toxicological concern detected were Ni (craft=14.37 µg/L; mainstream=10.41 µg/L; WHO limit 0.02 mg/l), Mn (craft=195 µg/L; mainstream= 155.48 µg/L; WHO limit not established), Hg (craft=0.02 µg/L; mainstream=0.03 µg/L; WHO limit not established), Cu (craft=41.67 µg/L; mainstream=56.36 µg/L; WHO limit 2 mg/l), Cr (craft=2.28 µg/L; mainstream=3.50 µg/L; WHO limit 0.05 mg/l) and As (craft= 1.01 µg/L; mainstream=3.20 µg/L; WHO limit 0.01 mg/l). Again, these levels are well below the toxicological limits established by the WHO, confirming beer as an alcoholic drink with a safe toxicological profile [14].

**Fig. 3:**
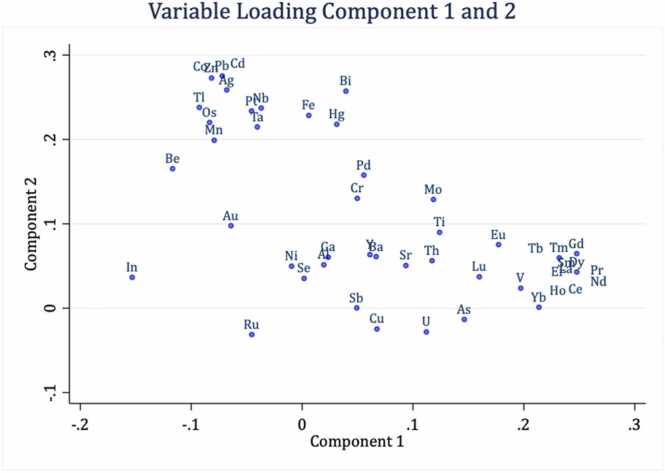
Principal Component Analysis model developed to explore the interdependence among elements with a 43 × 50 data matrix. Filtering the components which an eigenvalue larger than 1 (Kaiser criterion), we obtain 11 components explaining 0.85% of the total variance.

A Principal Component Analysis (PCA) model was developed to explore the interdependence among elements with a 43 × 50 data matrix. Filtering the components which an eigenvalue larger than 1 (Kaiser criterion), we obtain 11 components explaining 0.85% of the total variance. We plot the first and second components (see Fig. 3), which jointly explain 45% of the total variance. Some elements tend to cluster together, with two main clusters strongly opposed to one another. Thus, Zn, Pb, Co, Cd, Tl, Mn, Ta, Nb Pt, Bi, Hg and Fe cluster together, in opposition to Tm, Gd, Dy, Pr, Nd, La Yb, Er, V, Pm, Sb, Ce and Pr. Another cluster is formed by Ni, Al, Se and Ga. These clusters partially overlap with those described by Wyrzykowska et al. [66]. Although a different array and lesser number of elements were explored in their analysis, the first cluster described includes Tl, Bi, Co, Cd and Hg, which also appear in our first cluster. For them, the Co-Cd correlation derives from Co powder use in hard metal container processing, for enamels and pigments. To this we could add Fe, Zn and Pb, which can also derive from processing interferences with the brewing material, such as the Zn coating over ferrous materials and the use of Pb in old machinery. On the contrary, the clustering of Mn-V found by Wyrzykowska et al. [66] does not correlate with our findings, where these two metals stand in opposition.

## 4 Conclusion

The expansion of craft beers requires a specific focus on their quality and an in-depth exploration of their differences from mainstream beers. This paper shows that differences exist between craft and mainstream beers in all aspects under analysis, including mycotoxin profile, pesticide and contaminant residues, and elemental composition. Mainstream beers presented higher concentrations of mycotoxins than craft beers, which were unproblematic in this regard, although all samples were within the legal limits established for other alcoholic drinks. However, craft beers presented higher average concentrations of pesticide residues than their mainstream counterparts. This is most probably because the processing factors such as filtration and pasteurization employed by mainstream breweries can remove residues from the final product, while craft beers tend not to filter or pasteurize their products. Therefore, the health-associated claims about higher naturalness and quality of craft beers can be doubted in the case of pesticide residues. In this regard, it must be highlighted that the only beer without residues was certified organic. This shows that in this case certification might ensure consumers that they are drinking a beer devoid of residues, a hypothesis that requires further research comparing organic and mainstream beers.

Finally, the elemental composition of craft and mainstream beers differed, in both the order and concentration of different minor and trace elements analysed. Statistically significant differences were found in the concentration of various elements, with several orders of difference between some of them. The explanation for these differences rests on both endogenous factors (mainly raw materials and water) and exogenous (contamination from external sources and brewery machinery). Nonetheless, the levels of most elements remained within the ranges reported in the literature, and no elements of toxicological concern showed high levels.

These results are of potential interest to brewers, given that the presence of these compounds has an impact on beer quality and processing, from brewing to storage. Information on beer’s nutritional and toxicological profile is also important for consumers, reassuring them that both mainstream and craft beers are safe alcoholic beverages that largely comply with legal requirements. Nonetheless, specific Maximum Residue Limits are lacking for the occurrence of mycotoxins, pesticides and toxic metals in beer set by the European Union and internationally. This clearly hinders the development of literature and research in this area. The establishment of such limits would therefore be advisable, given that beer drinkers must be protected from consuming potentially contaminated beers. This study encountered some limitations, mainly the small sample size (N = 43) the local geographical scope of craft beers in the Canary Islands. Moreover, the fact that most craft beers are ales and most mainstream beers are lagers, poses challenges in interpreting whether differences derive from production method or beer style. Further research should focus on specific styles that may pose challenges, such as wheat beers.

## CRediT authorship contribution statement

Conceptualization: E.P.D. and P.A.G. Methodology and Formal analysis: P.A.B., O.P.L., I.H.T. and. A.C.A.D. Validation: O.P.L. and. A.C.A.D. Investigation: E.P.D and P.A.G. Funding acquisition: E.P.D. Drafting of manuscript by P.A.G. Review and comments by E.P.D., O.P.L., I.H.T. and. A.C.A.D. All authors read and approved the final manuscript.

## Declaration of Competing Interest

The authors declare that they have no known competing financial interests or personal relationships that could have appeared to influence the work reported in this paper.

## Data Availability

Data will be made available on request.
