# Supplementary material for: Comparative analysis of mycotoxin, pesticide, and elemental content of Canarian craft and Spanish mainstream beers
Source: Toxicol Rep. 2023 Mar 20;10:389–99. doi: 10.1016/j.toxrep.2023.03.003 (PMC10038769; doi:10.1016/j.toxrep.2023.03.003)
Supplement: Tables S1 — Supplementary material [file mmc1.docx]

**Table S1. List of pesticides and POPs analysed with their category of use, analysis technique, retention time and mass spectrometric conditions**

| **Compound** | **Category ^a^** | **Technique** ^b^ | **Retention time (min)** | **Polarity** | **Quantification** | | **Confirmation** | | **Fragmentor** |
| --- | --- | --- | --- | --- | --- | --- | --- | --- | --- |
|  |  |  |  |  | **MRM transition (m/z)** | **CE (eV)** | **MRM transition (m/z)** | **CE (eV)** |  |
| 4.4'-Dichlorobenzophenone (metabolite of dicofol) | Met | GC | 9.97 | Positive | 250.0 -> 139.0 | 15 | 250.0 -> 215.0 | 5 | 70 |
| 4.4'-Dicofol | OCP | GC | 13.14 | Positive | 251.0 -> 111.0 | 15 | 251.0 -> 139.0 | 45 | 70 |
| Abamectine | I, A, AH | LC | 10.93 | Positive | 890.5 -> 567.1 | 50 | 895.5 -> 751.4 | 45 | 160 |
| Acephate | I | LC | 1.90 | Positive | 184.0 -> 143.0 | 0 | 184.0 -> 95.0 | 20 | 70 |
| Acetamiprid | I | LC | 4.39 | Positive | 223.1 -> 126.0 | 45 | 223.1 -> 90.0 | 27 | 140 |
| Acrinathrin | I, A | LC | 10.65 | Positive | 559.0 -> 208.0 | 30 | 559.0 -> 181.0 | 10 | 76 |
| Aldicarb | I | LC | 5.09 | Positive | 116.0 -> 89.1 | 4 | 208.0 -> 116.0 | 0 | 100 |
| Aldicarb sulfone | Met | LC | 3.15 | Positive | 240.1 -> 76.0 | 13 | 223.1 -> 86.1 | 13 | 120 |
| Atrazine | H | LC | 6.70 | Positive | 216.0 -> 68.1 | 15 | 216.0 -> 103.8 | 30 | 130 |
| Azinphos methyl | I | LC | 7.22 | Positive | 318.0 -> 132.1 | 15 | 318.0 -> 261.0 | 8 | 90 |
| Azoxystrobin | F | LC | 7.53 | Positive | 404.1 -> 344.1 | 8 | 404.1 -> 329.1 | 32 | 110 |
| Benalaxyl | F | LC | 8.90 | Positive | 326.2 -> 148.0 | 20 | 326.2 -> 208.0 | 12 | 90 |
| Bendiocarb | I | LC | 5.84 | Positive | 224.1 -> 166.9 | 8 | 224.1 -> 108.9 | 30 | 100 |
| Bifenthrin | I | GC | 13.84 | Positive | 181.2 -> 165.2 | 25 | 181.2 -> 115.0 | 60 | 70 |
| Bitertanol | F | LC | 9.17 | Positive | 338.2 -> 70.0 | 5 | 338.2 -> 269.2 | 4 | 100 |
| Boscalid (formely nicobifen) | F | GC | 16.53 | Positive | 342.0 -> 140.0 | 15 | 342.0 -> 112.0 | 45 | 70 |
| Bromopropylate | A | GC | 13.84 | Positive | 341.0 -> 183.0 | 15 | 341.0 -> 157.0 | 45 | 70 |
| Bromuconazole (two isomers) | F | LC | 8.09/8.67 | Positive | 378.0 -> 159.0 | 35 | 376.0 -> 159.0 | 32 | 150 |
| Bupirimate | F | LC | 8.30 | Positive | 317.2 -> 108.1 | 28 | 317.2 -> 166.1 | 18 | 100 |
| Buprofezin | I | LC | 9.79 | Positive | 306.1 -> 201.0 | 12 | 306.1 -> 116.0 | 12 | 140 |
| Cadusafos (ebufos) | I, AH | LC | 9.33 | Positive | 271.1 -> 159.0 | 22 | 271.1 -> 131.0 | 16 | 100 |
| Carbaryl | I | LC | 6.16 | Positive | 202.1 -> 145.1 | 4 | 202.1 -> 127.1 | 28 | 90 |
| Carbofuran | I, AH | LC | 5.88 | Positive | 222.1 -> 123.1 | 20 | 222.1 -> 165.1 | 30 | 80 |
| Carbofuran-3-hydroxy | Met | LC | 4.37 | Positive | 238.1 -> 163.1 | 10 | 238.1 -> 181.1 | 10 | 110 |
| Chlorantraniliprole | I | LC | 7.27 | Positive | 483.9 -> 452.9 | 16 | 483.9 -> 285.9 | 8 | 105 |
| Chlorfenapyr | I, A | GC | 11.97 | Positive | 247.0 -> 227.0 | 15 | 328.0 -> 247.0 | 20 | 70 |
| Chlorfenvinphos | I | LC | 8.98 | Positive | 358.9 -> 155.1 | 8 | 361.1 -> 154.9 | 34 | 105 |
| Chlorobenzilate | A | GC | 12.09 | Positive | 251.0 -> 139.0 | 15 | 251.0 -> 111.0 | 40 | 70 |
| Chlorpropham | H | GC | 7.05 | Positive | 213.0 -> 127.0 | 15 | 153.0 -> 90.0 | 10 | 70 |
| Chlorpyrifos | I | GC | 9.88 | Positive | 314.0 -> 258.0 | 15 | 314.0 -> 286.0 | 5 | 70 |
| Chlorpyrifos methyl | I | GC | 9.07 | Positive | 286.0 -> 93.0 | 25 | 286.0 -> 271.0 | 15 | 70 |
| Chlorthal dimethyl | H | GC | 9.98 | Positive | 300.9 -> 166.9 | 55 | 300.9 -> 222.9 | 25 | 70 |
| Clofentezine | A | LC | 9.12 | Positive | 303.1 -> 138.0 | 12 | 303.1 -> 102.0 | 40 | 120 |
| Clothianidin | I | LC | 4.09 | Positive | 250.0 -> 169.0 | 8 | 250.0 -> 131.9 | 8 | 100 |
| Coumachlor | R | LC | 8.55 | Positive | 343.1 -> 162.8 | 15 | 343.1 -> 285.0 | 15 | 120 |
| Coumaphos | I, A | LC | 8.92 | Positive | 363.0 -> 227.0 | 30 | 363.0 -> 306.9 | 15 | 120 |
| Cyazofamid | F | LC | 8.42 | Positive | 325.0 -> 108.0 | 20 | 325.0 -> 261.1 | 15 | 90 |
| Cyflufenamid | F | LC | 9.12 | Positive | 413.1 -> 223.1 | 23 | 413.1 -> 295.1 | 33 | 70 |
| Cyfluthrin (sum of four isomers) | I | GC | 16.11/16.2/16.27/16.31 | Positive | 226.0 -> 206.0 | 25 | 226.0 -> 199.0 | 10 | 70 |
| Cyhalothrin (lambda isomer) | I | LC | 10.43 | Positive | 467.0 -> 225.0 | 10 | 467.0 -> 141.0 | 46 | 66 |
| Cymoxanil | F | LC | 4.64 | Positive | 199.1 -> 128.0 | 4 | 199.1 -> 110.9 | 12 | 90 |
| Cypermethrin (sum of four isomers) | I | GC | 16.42/16.51/16.60/16.62 | Positive | 163.0 -> 127.0 | 15 | 163.0 -> 109.0 | 5 | 70 |
| Cyproconazole (two isomers) | F | LC | 7.97/8.18 | Positive | 292.2 -> 70.2 | 18 | 292.2 -> 125.1 | 24 | 100 |
| Cyprodinil | F | GC | 10.33 | Positive | 224.0 -> 118.0 | 45 | 224.0 -> 104.0 | 25 | 70 |
| Deltamethrin | I, A | GC | 18.06 | Positive | 181.0 -> 152.1 | 15 | 251.0 -> 172.0 | 25 | 70 |
| Demeton-S-methyl | I, A | LC | 5.92 | Positive | 230.9 -> 88.9 | 5 | 230.9 -> 61.0 | 30 | 50 |
| Demeton-S-methyl-sulfone (Dioxydemeton) | I, A | LC | 3.54 | Positive | 263.0 -> 169.0 | 24 | 263.0 -> 109.0 | 12 | 120 |
| Diazinon | I | GC | 8.21 | Positive | 304.0 -> 179.0 | 15 | 137.1 -> 84.0 | 20 | 70 |
| Dichlofluanid | F | GC | 9.68 | Positive | 224.0 -> 123.0 | 10 | 226.0 -> 123.0 | 15 | 70 |
| Dichloran | F, MB, WP | GC | 7.75 | Positive | 206.0 -> 176.0 | 25 | 206.0 -> 148.0 | 10 | 70 |
| Diethathyl ethyl | H | LC | 8.66 | Positive | 312.2 -> 238.1 | 15 | 312.2 -> 162.0 | 30 | 120 |
| Diethofencarb | F, MB, WP | LC | 7.52 | Positive | 268.2 -> 226.1 | 5 | 268.2 -> 152.0 | 20 | 110 |
| Difenoconazole | F, MB, WP | LC | 9.35 | Positive | 406.1 -> 250.9 | 28 | 406.1 -> 337.0 | 16 | 176 |
| Diflubenzuron | I | LC | 8.55 | Positive | 311.0 -> 158.0 | 32 | 311.0 -> 141.0 | 8 | 90 |
| Diflufenican | H | LC | 9.44 | Positive | 395.1 -> 266.0 | 24 | 395.1 -> 246.0 | 36 | 150 |
| Dimethenamide | H | LC | 7.65 | Positive | 276.1 -> 244.1 | 10 | 276.1 -> 168.1 | 20 | 125 |
| Dimethoate | I | LC | 4.32 | Positive | 230.0 -> 198.8 | 16 | 230.0 -> 125.0 | 0 | 70 |
| Dimethomorph (two isomers) | F, MB, WP | LC | 7.68/7.94 | Positive | 388.1 -> 301.1 | 20 | 388.1 -> 165.1 | 32 | 180 |
| Diniconazole-M | F, MB, WP | LC | 9.26 | Positive | 326.1 -> 70.0 | 28 | 328.1 -> 70.0 | 28 | 110 |
| Dinocap | F, MB, WP | LC | 10.45 | Negative | 295.4 -> 193.0 | 30 | 295.4 -> 163.0 | 40 | 150 |
| Diphenylamine | PHP | LC | 4.24 | Positive | 170.0 -> 65.0 | 65 | 170.0 -> 93.0 | 40 | 200 |
| Endosulfan alfa | OCP | GC | 11.18 | Positive | 241.0 -> 206.0 | 10 | 195.0 -> 160.0 | 15 | 70 |
| Endosulfan beta | OCP | GC | 12.19 | Positive | 195.0 -> 125.0 | 25 | 195.0 -> 159.0 | 10 | 70 |
| EPN | I, A | LC | 9.38 | Positive | 324.0 -> 157.0 | 25 | 324.0 -> 296.0 | 14 | 88 |
| Epoxiconazole | F | LC | 8.40 | Positive | 330.0 -> 100.9 | 50 | 330.0 -> 120.9 | 24 | 120 |
| Esfenvalerate | I | GC | 17.54 | Positive | 167.0 -> 125.1 | 45 | 225.0 -> 119.0 | 15 | 70 |
| Ethion (diethion) | I, A | LC | 9.95 | Positive | 385.0 -> 199.0 | 5 | 385.0 -> 171.0 | 10 | 100 |
| Ethofumesate | H | GC | 9.54 | Positive | 286.0 -> 207.0 | 5 | 286.0 -> 161.0 | 20 | 70 |
| Ethoprophos | I, AH | LC | 8.33 | Positive | 243.1 -> 97.0 | 30 | 243.1 -> 130.9 | 15 | 90 |
| Etofenprox | I, A | LC | 11.13 | Positive | 394.0 -> 359.0 | 10 | 394.0 -> 135.1 | 40 | 66 |
| Etoxazole | A | LC | 10.27 | Positive | 360.1 -> 304.0 | 28 | 360.1 -> 113.0 | 26 | 160 |
| Famoxadone | H | LC | 9.01 | Positive | 392.1 -> 330.9 | 5 | 392.1 -> 238.1 | 12 | 110 |
| Fenamidone | F | LC | 7.67 | Positive | 312.0 -> 92.2 | 28 | 312.0 -> 236.1 | 14 | 100 |
| Fenamiphos | I, AH | LC | 8.57 | Positive | 304.1 -> 217.1 | 20 | 304.1 -> 202.0 | 36 | 120 |
| Fenamiphos sulfone | Met | LC | 6.19 | Positive | 336.1 -> 308.1 | 23 | 336.1 -> 188.0 | 31 | 120 |
| Fenamiphos sulfoxide | Met | LC | 6.03 | Positive | 320.1 -> 233.0 | 20 | 320.1 -> 108.1 | 44 | 120 |
| Fenarimol | F, MB, WP | GC | 15.02 | Positive | 139.0 -> 75.0 | 30 | 139.0 -> 111.0 | 15 | 70 |
| Fenazaquin | A | LC | 10.65 | Positive | 307.2 -> 161.1 | 25 | 307.2 -> 131.0 | 16 | 130 |
| Fenbuconazole | F, V | LC | 8.51 | Positive | 337.1 -> 70.0 | 40 | 337.1 -> 125.1 | 33 | 160 |
| Fenbutatin oxide | I, A | LC | 11.51 | Positive | 519.0 -> 90.9 | 65 | 519.0 -> 197.0 | 55 | 180 |
| Fenitrothion | I | GC | 9.50 | Positive | 277.0 -> 109.0 | 15 | 277.0 -> 260.0 | 5 | 70 |
| Fenoxycarb | I | LC | 8.63 | Positive | 302.1 -> 88.0 | 10 | 302.1 -> 116.1 | 20 | 110 |
| Fenpropathrin | I, A | LC | 10.37 | Positive | 367.2 -> 125.0 | 16 | 350.2 -> 125.0 | 16 | 72 |
| Fenpropimorph | F | LC | 7.39 | Positive | 304.3 -> 147.1 | 30 | 304.3 -> 130.0 | 25 | 120 |
| Fenpyroximate | A | LC | 10.42 | Positive | 422.2 -> 366.2 | 12 | 422.2 -> 135.0 | 36 | 160 |
| Fenthion | I, A | LC | 8.82 | Positive | 279.0 -> 168.8 | 8 | 279.0 -> 247.1 | 18 | 98 |
| Fenthion oxon | Met | LC | 7.26 | Positive | 263.1 -> 231.2 | 16 | 263.1 -> 216.0 | 24 | 120 |
| Fenthion oxon sulfone | Met | LC | 4.61 | Positive | 295.0 -> 217.0 | 15 | 295.0 -> 104.2 | 20 | 110 |
| Fenthion oxon sulfoxide | Met | LC | 4.46 | Positive | 279.0 -> 104.1 | 20 | 279.0 -> 264.2 | 28 | 110 |
| Fenthion sulfone | Met | LC | 6.32 | Positive | 311.0 -> 125.0 | 28 | 311.0 -> 109.0 | 22 | 140 |
| Fenthion sulfoxide | Met | LC | 6.10 | Positive | 295.0 -> 280.0 | 15 | 295.0 -> 108.9 | 30 | 140 |
| Fenvalerate | I | GC | 17.34 | Positive | 167.0 -> 125.1 | 45 | 225.0 -> 119.0 | 15 | 70 |
| Fipronil | I, V | LC | 8.61 | Negative | 435.0 -> 330.0 | 26 | 435.0 -> 249.9 | 12 | 116 |
| Fipronil sulfide | Met | GC | 10.44 | Positive | 351.0 -> 255.0 | 20 | 420.0 -> 351.0 | 35 | 70 |
| Fluazinam | F | LC | 9.94 | Negative | 462.9 -> 416.0 | 10 | 462.9 -> 398.0 | 9 | 140 |
| Flubendiamide | I | LC | 8.75 | Positive | 408.0 -> 274.0 | 15 | 408.0 -> 256.0 | 30 | 120 |
| Flucythrinate (two isomers) | I, A | GC | 16.63/16.82 | Positive | 199.1 -> 107.1 | 25 | 156.9 -> 107.1 | 15 | 70 |
| Fludioxonil | F | LC | 7.71 | Negative | 247.0 -> 180.0 | 62 | 247.0 -> 125.9 | 32 | 152 |
| Flufenoxuron | I, A | LC | 10.30 | Positive | 489.1 -> 158.0 | 20 | 489.1 -> 140.9 | 56 | 110 |
| Fluopyram | F | LC | 8.18 | Positive | 397.0 -> 173.0 | 40 | 397.0 -> 145.0 | 50 | 150 |
| Fluquinconazole | F | LC | 8.21 | Positive | 376.0 -> 307.1 | 56 | 376.0 -> 108.0 | 24 | 140 |
| Flusilazole | F, MB, WP | LC | 8.59 | Positive | 316.1 -> 247.1 | 20 | 316.1 -> 165.0 | 15 | 160 |
| Flutolanil | F, MB, WP | LC | 7.87 | Positive | 324.1 -> 262.1 | 16 | 324.1 -> 242.1 | 24 | 130 |
| Flutriafol | F | LC | 6.77 | Positive | 302.1 -> 70.1 | 16 | 302.1 -> 122.9 | 28 | 90 |
| Fluvalinate tau | I, A | LC | 10.81 | Positive | 503.0 -> 208.0 | 10 | 503.0 -> 181.0 | 26 | 50 |
| Fonofos | I | GC | 8.18 | Positive | 246.0 -> 109.0 | 5 | 246.0 -> 137.0 | 15 | 70 |
| Fosthiazate | AH, V | LC | 6.46 | Positive | 284.0 -> 104.0 | 20 | 284.0 -> 227.8 | 8 | 110 |
| Hexaconazole | F, MB, WP | LC | 9.06 | Positive | 316.1 -> 70.1 | 20 | 314.1 -> 70.1 | 20 | 95 |
| Hexaflumuron | I | LC | 9.52 | Negative | 459.1 -> 439.0 | 18 | 459.1 -> 276.1 | 8 | 100 |
| Hexythiazox | A | LC | 10.10 | Positive | 353.1 -> 227.9 | 8 | 353.1 -> 168.1 | 24 | 120 |
| Imidacloprid | I | LC | 4.07 | Positive | 256.0 -> 208.9 | 12 | 256.0 -> 175.0 | 12 | 110 |
| Indoxacarb | I | LC | 9.42 | Positive | 528.1 -> 293.1 | 48 | 528.1 -> 202.8 | 10 | 140 |
| Iprodione | F, MB, WP | GC | 13.63 | Positive | 314 -> 56.0 | 20 | 314.0 -> 245.0 | 10 | 70 |
| Iprovalicarb | F | LC | 8.17 | Positive | 321.2 -> 119.0 | 15 | 321.2 -> 202.9 | 0 | 108 |
| Isocarbophos | I | GC | 10.34 | Positive | 230.0 -> 155.0 | 25 | 230.0 -> 198.0 | 10 | 70 |
| Isofenphos methyl | I | LC | 8.75 | Positive | 332.1 -> 230.9 | 44 | 332.1 -> 120.9 | 10 | 100 |
| Isoprothiolane | F, MB, WP | LC | 7.87 | Positive | 291.1 -> 231.1 | 30 | 291.1 -> 189.0 | 12 | 100 |
| Kresoxim methyl | F | LC | 8.74 | Positive | 314.1 -> 116.0 | 24 | 314.1 -> 223.0 | 15 | 98 |
| Linuron | F | LC | 7.48 | Positive | 249.0 -> 160.1 | 20 | 249.0 -> 182.3 | 8 | 120 |
| Lufenuron | I | LC | 10.00 | Negative | 509.0 -> 339.0 | 5 | 509.0 -> 326.1 | 15 | 90 |
| Malaoxon | I | LC | 5.99 | Positive | 315.1 -> 127.2 | 12 | 315.1 -> 99.1 | 12 | 120 |
| Malathion | I | LC | 7.87 | Positive | 348.0 -> 126.7 | 15 | 348.0 -> 285.0 | 8 | 100 |
| Mandipropamid | F | LC | 7.85 | Positive | 412.1 -> 328.0 | 8 | 412.1 -> 356.1 | 4 | 130 |
| Mefenoxam (metalaxyl-M) | F | LC | 6.90 | Positive | 280.0 -> 220.0 | 10 | 280.0 -> 192.0 | 15 | 110 |
| Mepanipyrim | F, MB, WP | LC | 8.14 | Positive | 224.1 -> 106.0 | 30 | 224.1 -> 77.0 | 25 | 120 |
| Metaflumizone | I | LC | 9.87 | Negative | 505.0 -> 302.0 | 10 | 541.0 -> 302.0 | 20 | 110 |
| Metalaxyl | F | GC | 9.25 | Positive | 234.0 -> 146.0 | 20 | 249.0 -> 146.0 | 5 | 70 |
| Metaldehyde | M | LC | 3.89 | Positive | 194.1 -> 61.9 | 5 | 194.1 -> 106.0 | 5 | 50 |
| Metconazole | F | LC | 9.11 | Positive | 320.1 -> 70.1 | 33 | 322.1 -> 70.1 | 24 | 110 |
| Methamidophos | I, A | LC | 1.26 | Positive | 142.0 -> 94.0 | 12 | 142.0 -> 125.0 | 12 | 85 |
| Methidathion | I, A | LC | 7.05 | Positive | 320.1 -> 144.8 | 8 | 320.1 -> 85.0 | 30 | 82 |
| Methiocarb | I, A, M | LC | 7.62 | Positive | 226.1 -> 169.0 | 12 | 226.1 -> 121.1 | 4 | 90 |
| Methiocarb sulfone | Met | LC | 4.56 | Positive | 258.1 -> 122.1 | 22 | 258.1 -> 201.1 | 8 | 100 |
| Methiocarb sulfoxide | Met | LC | 4.24 | Positive | 242.0 -> 122.0 | 28 | 242.0 -> 185.0 | 22 | 90 |
| Methomyl | I, A, AH | LC | 3.44 | Positive | 163.1 -> 88.0 | 8 | 163.1 -> 106.0 | 5 | 80 |
| Methomyl oxime | Met | LC | 2.43 | Positive | 106.2 -> 58.1 | 10 | 106.2 -> 42.2 | 40 | 70 |
| Methoxyfenozide | I | LC | 7.94 | Positive | 369.2 -> 149.0 | 10 | 369.2 -> 313.1 | 0 | 80 |
| Metrafenone | F | LC | 9.20 | Positive | 409.1 -> 209.1 | 8 | 411.2 -> 209.1 | 12 | 120 |
| Mevinphos (phosdrin) (two isomers) | I, A | LC | 4.35/4.85 | Positive | 225.0 -> 193.1 | 0 | 225.0 -> 127.0 | 12 | 65 |
| Monocrotophos | I | LC | 3.69 | Positive | 224.1 -> 126.8 | 15 | 224.1 -> 98.1 | 12 | 100 |
| Myclobutanil | F, MB, WP | LC | 8.05 | Positive | 289.1 -> 70.1 | 16 | 289.1 -> 125.1 | 32 | 110 |
| N.N-Dimethyl-N'-p-tolylsulphamide (DMST, metabolite of tolylfluanid) | Met | LC | 6.01 | Positive | 215.1 -> 106.1 | 4 | 215.1 -> 151.1 | 10 | 90 |
| N.N-dimethylformamidine (DMF, metabolite of amitraz) | Met ^g^ | LC | 5.40 | Positive | 149.9 -> 105.8 | 30 | 149.9 -> 122.9 | 15 | 100 |
| Nuarimol | F, MB, WP | LC | 7.57 | Positive | 315.0 -> 252.0 | 30 | 315.0 -> 81.1 | 28 | 80 |
| Ofurace | F, MB, WP | LC | 5.94 | Positive | 282.0 -> 159.9 | 20 | 282.0 -> 147.9 | 30 | 100 |
| Omethoate | I, A | LC | 2.56 | Positive | 214.1 -> 124.8 | 22 | 214.1 -> 183.0 | 5 | 84 |
| Oxadixyl | F, MB, WP | LC | 5.41 | Positive | 279.1 -> 219.2 | 5 | 279.1 -> 132.3 | 32 | 110 |
| Oxamyl | I, A, AH | LC | 3.24 | Positive | 237.1 -> 72.0 | 12 | 237.1 -> 90.0 | 5 | 70 |
| Oxamyl oxime | Met | LC | 2.76 | Positive | 163.3 -> 72.1 | 15 | 163.3 -> 90.0 | 10 | 70 |
| Oxyfluorfen | H | GC | 11.64 | Positive | 252.0 -> 146.0 | 20 | 252.0 -> 196.0 | 40 | 70 |
| Paclobutrazol | H | LC | 7.86 | Positive | 294.1 -> 70.1 | 16 | 294.1 -> 125.2 | 36 | 115 |
| Paraoxon methyl | I | GC | 8.94 | Positive | 230.0 -> 106.0 | 20 | 230.0 -> 136.0 | 5 | 70 |
| Parathion ethyl | I | GC | 9.90 | Positive | 290.9 -> 109.0 | 30 | 138.9 -> 109.0 | 5 | 70 |
| Parathion methyl | I | GC | 9.09 | Positive | 263.0 -> 109.0 | 15 | 263.0 -> 79.0 | 30 | 70 |
| Penconazole | F, MB, WP | LC | 8.80 | Positive | 284.1 -> 70.1 | 30 | 285.1 -> 195.0 | 15 | 70 |
| Pencycuron | F, MB, WP | LC | 9.26 | Positive | 329.1 -> 125.1 | 24 | 329.1 -> 217.9 | 12 | 160 |
| Pendimethalin | H | LC | 10.13 | Positive | 282.2 -> 212.2 | 10 | 282.2 -> 194.1 | 17 | 80 |
| Permethrin (two isomers) | I, A | GC | 15.54/16.67 | Positive | 183.1 -> 168.1 | 15 | 183.1 -> 165.1 | 10 | 70 |
| Phosalone | I, A | LC | 9.13 | Positive | 385.1 -> 182.0 | 20 | 385.1 -> 110.9 | 55 | 80 |
| Phosmet | I, A | LC | 7.27 | Positive | 318.0 -> 159.9 | 16 | 318.0 -> 133.0 | 40 | 90 |
| Phosmet oxon | Met | LC | 5.32 | Positive | 302.0 -> 160.0 | 10 | 302.0 -> 77.0 | 55 | 60 |
| Phthalimide (metabolite folpet) | Met ^h^ | GC | 5.81 | Positive | 104.0 -> 50.0 | 25 | 147.0 -> 76.0 | 25 | 70 |
| Pirimicarb | I | LC | 5.17 | Positive | 239.1 -> 72.1 | 20 | 239.1 -> 182.1 | 12 | 100 |
| Pirimiphos ethyl | I, A | LC | 9.86 | Positive | 334.1 -> 182.1 | 23 | 334.1 -> 198.1 | 25 | 100 |
| Pirimiphos methyl | I, A | LC | 9.08 | Positive | 306.1 -> 108.1 | 32 | 306.1 -> 164.0 | 20 | 100 |
| Prochloraz | F, MB, WP | LC | 9.03 | Positive | 376.0 -> 308.0 | 10 | 376.0 -> 70.1 | 20 | 100 |
| Procymidone | F, MB, WP | GC | 10.77 | Positive | 283.0 -> 67.0 | 40 | 283.0 -> 68.0 | 25 | 70 |
| Profenofos | I, A | LC | 9.67 | Positive | 375.0 -> 304.8 | 20 | 373.0 -> 302.8 | 20 | 100 |
| Propargite | A | LC | 10.29 | Positive | 368.2 -> 231.1 | 4 | 368.2 -> 175.0 | 12 | 88 |
| Propiconazole | A | LC | 8.96 | Positive | 342.0 -> 69.0 | 21 | 342.0 -> 159.0 | 39 | 90 |
| Propoxur | I | LC | 5.81 | Positive | 210.1 -> 111.0 | 12 | 210.1 -> 168.1 | 0 | 70 |
| Propyzamide (pronamide) | H | LC | 7.86 | Positive | 256.1 -> 190.0 | 16 | 256.1 -> 173.0 | 25 | 90 |
| Proquinazid | F | LC | 10.53 | Positive | 372.9 -> 331.0 | 20 | 372.9 -> 289.0 | 5 | 100 |
| Prothioconazole-desthio | Met | LC | 8.44 | Positive | 312.0 -> 70.1 | 22 | 312.0 -> 125.0 | 18 | 100 |
| Prothiophos | F | GC | 11.43 | Positive | 162.0 -> 63.1 | 5 | 266.9 -> 221.0 | 20 | 70 |
| Pyraclostrobin | F | LC | 9.07 | Positive | 388.1 -> 193.8 | 8 | 388.1 -> 163.1 | 28 | 120 |
| Pyrazophos | F, MB, WP | LC | 9.15 | Positive | 374.1 -> 222.1 | 23 | 374.1 -> 194.0 | 32 | 100 |
| Pyridaben | I, A | LC | 10.68 | Positive | 365.2 -> 309.0 | 8 | 309.1 -> 147.0 | 16 | 96 |
| Pyridaphenthion | I, A | LC | 8.06 | Positive | 341.0 -> 189.0 | 22 | 341.0 -> 92.0 | 34 | 100 |
| Pyrimethanil | F | GC | 8.22 | Positive | 198.0 -> 118.0 | 40 | 198.0 -> 158.0 | 20 | 70 |
| Pyriproxifen | I | LC | 10.01 | Positive | 322.2 -> 96.0 | 12 | 322.2 -> 184.9 | 24 | 80 |
| Quinalphos | I, A | LC | 8.67 | Positive | 299.1 -> 96.9 | 20 | 299.1 -> 147.1 | 30 | 130 |
| Quinoxyfen | F | LC | 10.05 | Positive | 308.0 -> 197.0 | 32 | 308.0 -> 161.8 | 55 | 100 |
| Rotenone | I, R | LC | 8.58 | Positive | 395.1 -> 192.1 | 25 | 395.1 -> 213.1 | 20 | 150 |
| Simazine | I | LC | 5.79 | Positive | 202.4 -> 131.9 | 20 | 202.4 -> 68.1 | 30 | 120 |
| Spirodiclofen | A | LC | 10.44 | Positive | 411.1 -> 71.2 | 5 | 411.1 -> 313.0 | 15 | 110 |
| Spiromesifen | I | LC | 10.21 | Positive | 371.0 -> 273.0 | 25 | 273.0 -> 187.0 | 15 | 90 |
| Spirotetramat | I | LC | 8.26 | Positive | 374.2 -> 302.2 | 12 | 374.2 -> 216.1 | 36 | 150 |
| Spirotetramat-enol | Met | LC | 8.27 | Positive | 302.0 -> 216.0 | 20 | 302.0 -> 270.0 | 30 | 180 |
| Spiroxamine (two isomers) | F | GC | 9.02/9.47 | Positive | 100.0 -> 72.0 | 5 | 100.0 -> 58.0 | 10 | 70 |
| Tebuconazole | I, A | LC | 8.85 | Positive | 308.2 -> 70.2 | 22 | 308.2 -> 125.1 | 53 | 120 |
| Tebufenocide | I | LC | 8.60 | Positive | 353.1 -> 132.9 | 22 | 353.1 -> 297.1 | 0 | 98 |
| Tebufenpyrad | A | LC | 9.82 | Positive | 334.2 -> 117.0 | 47 | 334.2 -> 145.0 | 37 | 180 |
| Teflubenzuron | I | GC | 5.33 | Positive | 197.0 -> 135.0 | 25 | 197.0 -> 142.0 | 25 | 70 |
| Tefluthrin | I | GC | 8.34 | Positive | 177.0 -> 127.0 | 15 | 177.0 -> 87.0 | 35 | 70 |
| Telodrin (isobenzan) | I | GC | 10.10 | Positive | 310.8 -> 274.8 | 5 | 310.8 -> 240.8 | 25 | 70 |
| Terbufos | I, AH | GC | 8.09 | Positive | 231.0 -> 129.0 | 10 | 231.0 -> 97.0 | 20 | 70 |
| Terbuthylazine | H | LC | 7.65 | Positive | 230.0 -> 174.0 | 16 | 230.0 -> 96.0 | 28 | 100 |
| Tetrachlorvinphos | I | LC | 8.67 | Positive | 367.0 -> 127.0 | 16 | 364.9 -> 127.0 | 16 | 110 |
| Tetraconazole | F, H | LC | 8.36 | Positive | 372.0 -> 159.0 | 30 | 372.0 -> 70.1 | 20 | 100 |
| Tetradifon | A | GC | 14.34 | Positive | 158.9 -> 111.0 | 15 | 229.0 -> 201.0 | 20 | 70 |
| Tetramethrin | I | GC | 13.80 | Positive | 164.0 -> 77.0 | 30 | 164.0 -> 107.0 | 15 | 70 |
| Thiacloprid | I | LC | 4.76 | Positive | 253.0 -> 126.0 | 40 | 253.0 -> 90.0 | 16 | 140 |
| Thiamethoxam | I | LC | 3.56 | Positive | 292.0 -> 211.1 | 8 | 292.0 -> 132.0 | 22 | 80 |
| Thiodicarb | I | LC | 6.45 | Positive | 355.1 -> 88.1 | 8 | 355.1 -> 108.1 | 8 | 60 |
| Tolclofos methyl | F, MB, WP | GC | 9.15 | Positive | 265.0 -> 93.0 | 30 | 265.0 -> 220.0 | 25 | 70 |
| Tolylfluanid | F | GC | 10.56 | Positive | 238.0 -> 137.0 | 35 | 238.0 -> 91.0 | 35 | 70 |
| Triadimefon | F, MB, WP | LC | 7.97 | Positive | 294.1 -> 69.3 | 20 | 294.1 -> 197.2 | 15 | 100 |
| Triadimenol | F, MB, WP | LC | 8.18 | Positive | 296.1 -> 70.0 | 10 | 298.1 -> 70.0 | 10 | 80 |
| Triazophos (hostathion) | I, A | LC | 8.12 | Positive | 314.1 -> 162.0 | 19 | 314.1 -> 118.9 | 35 | 100 |
| Trichlorfon | I, AH, V | LC | 4.29 | Positive | 256.9 -> 109.0 | 12 | 258.9 -> 109.0 | 12 | 170 |
| Trifloxystrobin | F | LC | 9.44 | Positive | 409.1 -> 186.0 | 12 | 409.1 -> 145.0 | 52 | 110 |
| Triflumizole | F | LC | 9.49 | Positive | 346.1 -> 278.0 | 15 | 346.1 -> 73.0 | 4 | 80 |
| Triflumuron | I | LC | 9.13 | Positive | 359.0 -> 156.0 | 32 | 359.0 -> 139.0 | 8 | 120 |
| Trifluralin | H | GC | 7.17 | Positive | 306.0 -> 264.0 | 5 | 264.0 -> 160.0 | 15 | 70 |
| Triticonazole | F | LC | 8.34 | Positive | 318.1 -> 70.1 | 33 | 320.1 -> 70.1 | 16 | 110 |
| Vinclozolin | F, MB, WP | GC | 9.04 | Positive | 212.0 -> 145.0 | 45 | 212.0 -> 109.0 | 40 | 70 |
| Zoxamide | F | LC | 8.96 | Positive | 336.0 -> 187.1 | 25 | 187.1 -> 88.9 | 40 | 98 |
| Atrazine-d5 | P-IS | LC | 6.66 | Positive | 221.2 -> 179.0 | 15 | 221.2 -> 101.0 | 30 | 90 |
| Carbendazim-d3 | P-IS | LC | 3.45 | Positive | 195.1 -> 160.1 | 15 | 195.1 -> 131.9 | 30 | 100 |
| Cyromazine-d4 | P-IS | LC | 1.58 | Positive | 171.0 -> 86.0 | 15 | 171.0 -> 129.0 | 15 | 100 |
| Linuron-d3 | P-IS | LC | 7.45 | Positive | 255.1 -> 159.8 | 15 | 255.1 -> 185.0 | 15 | 100 |
| Pirimicarb-d6 | P-IS | LC | 5.12 | Positive | 245.2 -> 78.2 | 5 | 245.2 -> 185.1 | 15 | 70 |
| Chlorpyrifos-d10 | P-IS | GC | 9.85 | Positive | 324.0 -> 260.0 | 40 | 324.0 -> 195.0 | 55 | 70 |
| Diazinon-d10 | P-IS | GC | 8.21 | Positive | 314.0 -> 183.0 | 15 | 314.0 -> 199.0 | 5 | 70 |
| BDE 28 | PBDE | GC | 12.13 |  | 406.0 🡪 246.0 | 20 | 406.0 🡪 167.0 | 25 | 70 |
| BDE 47 | PBDE | GC | 14.21 |  | 326.0 🡪 138.0 | 45 | 484.0 🡪 324.0 | 25 | 70 |
| BDE 85 | PBDE | GC | 16.99 |  | 566.0 🡪 406.0 | 25 | 564.0 🡪 404.0 | 25 | 70 |
| BDE 99 | PBDE | GC | 16.18 |  | 566.0 🡪 406.0 | 25 | 564.0 🡪 404.0 | 25 | 70 |
| BDE 100 | PBDE | GC | 15.76 |  | 564.0 🡪 404.0 | 25 | 566.0 🡪 406.0 | 25 | 70 |
| BDE 153 | PBDE | GC | 17.96 |  | 644.0 🡪 484.0 | 30 | 486.0 🡪 377.0 | 30 | 70 |
| BDE 154 | PBDE | GC | 17.38 |  | 644.0 🡪 484.0 | 30 | 486.0 🡪 377.0 | 30 | 70 |
| BDE 183 | PBDE | GC | 19.98 |  | 563.6 🡪 454.7 | 40 | 561.6 🡪 454.7 | 40 | 70 |
| Aldrin | OCP | GC | 9.80 |  | 263.0 🡪 228.0 | 10 | 255.0 🡪 220.0 | 20 | 70 |
| Dichlorodiphenyldichloroethane (p,p’ DDD) | OCP | GC | 12.22 |  | 235.0 🡪 165.0 | 20 | 235.0 🡪 199.0 | 30 | 70 |
| Dichlorodiphenyldichloroethylene (p,p’ DDE) | OCP | GC | 11.48 |  | 318.0 🡪 176.0 | 60 | 318.0 🡪 248.0 | 15 | 70 |
| Dieldrin | OCP | GC | 11.58 |  | 263.0 🡪 228.0 | 15 | 277.0 🡪 241.0 | 15 | 70 |
| Endrin | OCP | GC | 11.94 |  | 263.0 🡪 193.0 | 35 | 245.0 🡪 173.0 | 25 | 70 |
| Heptachlor | OCP | GC | 9.21 |  | 272.0 🡪 237.0 | 15 | 274.0 🡪 239.0 | 15 | 70 |
| Hexachlorobenzene | OCP | GC | 7.70 |  | 284.0 🡪 214.0 | 40 | 284.0 🡪 249.0 | 25 | 70 |
| Hexachlorocyclohexane (alpha) | OCP | GC | 7.56 |  | 219.0 🡪 183.0 | 10 | 219.0 🡪 109.0 | 10 | 70 |
| Hexachlorocyclohexano (beta) | OCP | GC | 7.93 |  | 219.0 🡪 183.0 | 10 | 219.0 🡪 109.0 | 10 | 70 |
| Hexaclorociclohexano (delta) | OCP | GC | 8.43 |  | 219.0 🡪 183.0 | 15 | 219.0 🡪 109.0 | 45 | 70 |
| Hexachlorocyclohexane (gamma, lindane) | OCP | GC | 8.13 |  | 219.0 🡪 183.0 | 10 | 219.0 🡪 109.0 | 10 | 70 |
| Mirex | OCP | GC | 14.68 |  | 272.0 🡪 237.0 | 10 | 274.0 🡪 237.0 | 10 | 70 |
| Acenaphthene | PAH | GC | 6.10 |  | 153.0 🡪 152.0 | 25 | 153.0 🡪 151.0 | 35 | 70 |
| Acenaphthylene | PAH | GC | 5.89 |  | 152.0 🡪 151.0 | 25 | 152.0 🡪 126.0 | 30 | 70 |
| Anthracene | PAH | GC | 8.47 |  | 178.0 🡪 152.0 | 28 | 178.0 🡪 176.0 | 35 | 70 |
| Benzo[a]anthracene | PAH | GC | 13.74 |  | 228.0 🡪 226.0 | 40 | 228.0 🡪 202.0 | 35 | 70 |
| Benzo[b]fluoranthene | PAH | GC | 16.15 |  | 252.0 🡪 224.0 | 60 | 252.0 🡪 248.0 | 60 | 70 |
| Chrysene | PAH | GC | 14.01 |  | 228.0 🡪 226.0 | 40 | 228.0 🡪 227.0 | 25 | 70 |
| Fluoranthene | PAH | GC | 10.58 |  | 202.0 🡪 201.0 | 27 | 202.0 🡪 152.0 | 42 | 70 |
| Fluorene | PAH | GC | 6.75 |  | 165.0 🡪 163.0 | 40 | 165.0 🡪 139.0 | 32 | 70 |
| Naphthalene | PAH | GC | 4.42 |  | 128.0 🡪 102.0 | 25 | 128.0 🡪 127.0 | 15 | 70 |
| Phenanthrene | PAH | GC | 8.23 |  | 178.0 🡪 176.0 | 35 | 178.0 🡪 152.0 | 28 | 70 |
| Pyrene | PAH | GC | 11.04 |  | 202.0 🡪 200.0 | 45 | 202.0 🡪 201.0 | 27 | 70 |
| PCB 28 | PCB | GC | 8.94 |  | 256.0 🡪 186.0 | 25 | 256.0 🡪 151.0 | 50 | 70 |
| PCB 52 | PCB | GC | 9.49 |  | 292.0 🡪 222.0 | 25 | 292.0 🡪 220.0 | 25 | 70 |
| PCB 77 | PCB | GC | 11.65 |  | 292.0 🡪 222.0 | 25 | 292.0 🡪 220.0 | 25 | 70 |
| PCB 81 | PCB | GC | 11.47 |  | 292.0 🡪 222.0 | 25 | 292.0 🡪 220.0 | 25 | 70 |
| PCB 101 | PCB | GC | 10.98 |  | 326.0 🡪 256.0 | 30 | 328.0 🡪 256.0 | 30 | 70 |
| PCB 105 | PCB | GC | 12.56 |  | 326.0 🡪 256.0 | 30 | 328.0 🡪 256.0 | 30 | 70 |
| PCB 114 | PCB | GC | 12.29 |  | 326.0 🡪 256.0 | 30 | 328.0 🡪 256.0 | 30 | 70 |
| PCB 118 | PCB | GC | 12.15 |  | 326.0 🡪 256.0 | 30 | 328.0 🡪 256.0 | 30 | 70 |
| PCB 123 | PCB | GC | 12.01 |  | 326.0 🡪 256.0 | 30 | 328.0 🡪 256.0 | 30 | 70 |
| PCB 126 | PCB | GC | 13.14 |  | 326.0 🡪 256.0 | 30 | 328.0 🡪 256.0 | 30 | 70 |
| PCB 138 | PCB | GC | 12.97 |  | 360.0 🡪 290.0 | 25 | 360.0 🡪 288.0 | 25 | 70 |
| PCB 153 | PCB | GC | 12.47 |  | 360.0 🡪 290.0 | 25 | 360.0 🡪 288.0 | 25 | 70 |
| PCB 156 | PCB | GC | 13.86 |  | 360.0 🡪 290.0 | 25 | 360.0 🡪 288.0 | 25 | 70 |
| PCB 157 | PCB | GC | 13.96 |  | 360.0 🡪 290.0 | 25 | 360.0 🡪 288.0 | 25 | 70 |
| PCB 167 | PCB | GC | 13.45 |  | 360.0 🡪 290.0 | 25 | 360.0 🡪 288.0 | 25 | 70 |
| PCB 169 | PCB | GC | 14.52 |  | 360.0 🡪 290.0 | 25 | 360.0 🡪 288.0 | 25 | 70 |
| PCB 180 | PCB | GC | 14.14 |  | 394.0 🡪 324.0 | 30 | 394.0 🡪 322.0 | 30 | 70 |
| PCB 189 | PCB | GC | 15.15 |  | 394.0 🡪 324.0 | 30 | 394.0 🡪 322.0 | 30 | 70 |
| PCB 200 | P-IS | GC | 14.46 |  | 427.8 🡪 357.8 | 30 | 429.8 🡪 359.8 | 30 | 70 |

CE: Collision Energy

a POP – persistent organic pollutant, A – acaricide, MB – microbiocide, AH – anthelminthic, F – fungicide, H – herbicide, I – insecticide, R – plant growth regulator, WP – wood preservative, PHP – post-harvest preservative, M – Molluscicide, Met – metabolite, ^a^ BDE – Polybrominated diphenyl ethers, OCP – Organochlorine pesticides, PAH – Polycyclic aromatic hydrocarbon, PCB – Polychlorinated biphenyl, P-IS – Procedural Internal Standard

b Gas chromatography (GC) or liquid chromatography (LC), both coupled with tandem triple quadrupole mass spectrometry
