# Supplementary material for: Comparative analysis of mycotoxin, pesticide, and elemental content of Canarian craft and Spanish mainstream beers
Source: Toxicol Rep. 2023 Mar 20;10:389–99. doi: 10.1016/j.toxrep.2023.03.003 (PMC10038769; doi:10.1016/j.toxrep.2023.03.003)
Supplement: Tables S2 — Supplementary material [file mmc2.docx]

**Table S2. List of mycotoxins analysed together with their retention time and mass spectrometric conditions**

| **Compound** | **Retention time (min)** | **Polarity** | **Quantification** | | **Confirmation** | | **Fragmentor** |
| --- | --- | --- | --- | --- | --- | --- | --- |
|  |  |  | **MRM transition (m/z)** | **CE (eV)** | **MRM transition (m/z)** | **CE (eV)** |  |
| AFB1 | 9.705 | Positive | 313.1->241.0 | 41 | 313.1->285.0 | 21 | 100 |
| AFB2 | 9.215 | Positive | 315.1->287.0 | 21 | 315.1->258.9 | 29 | 100 |
| AFG1 | 8.712 | Positive | 329.1->243.0 | 25 | 329.1->200.0 | 41 | 100 |
| AFG2 | 8.194 | Positive | 331.1->313.0 | 21 | 331.1->245.0 | 25 | 100 |
| DON | 3.592 | Positive | 297.1->249.0 | 4 | 297.1->203.0 | 12 | 100 |
| FB1 | 12.001 | Positive | 722.4->352.4 | 37 | 722.4->334.4 | 37 | 100 |
| FB2 | 13.276 | Positive | 706.4->336.4 | 41 | 706.4->318.3 | 41 | 100 |
| HT2 | 11.334 | Positive | 442.2->263.0 | 9 | 442.2->215.0 | 13 | 100 |
| OTA | 13.136 | Positive | 404.1->358.0 | 10 | 404.1->238.9 | 25 | 100 |
| T2 | 12.234 | Positive | 484.3->215.0 | 9 | 484.3->305.0 | 8 | 100 |
| Zearalenone | 12.953 | Negative | 317.1->131.0 | 33 | 317.1->175.0 | 25 | 100 |

CE: Collision Energy
